# Genetic diversity and phylogenetic analyses of 11 cohorts of captive rhesus macaques from Chinese zoos

**DOI:** 10.7717/peerj.6957

**Published:** 2019-05-29

**Authors:** Qian Su, Yongfang Yao, Qin Zhao, Diyan Li, Meng Xie, Jiayun Wu, Anxiang Wen, Qin Wang, Guangxiang Zhu, Qingyong Ni, Mingwang Zhang, Huailiang Xu

**Affiliations:** 1 College of Life Science, Sichuan Agricultural University, Ya’an, Sichuan, China; 2 College of Animal Science and Technology, Sichuan Agricultural University, Chengdu, Sichuan, China

**Keywords:** Mitochondrial DNA, Rhesus macaque, *Macaca mulatta*, Genetic diversity, Phylogenetic analysis

## Abstract

Rhesus macaques are raised in almost every Chinese zoo due to their likeability and ease in feeding; however, little is yet known about the genetic diversity of rhesus macaques in captivity. In this study, a 475-base pair nucleotide sequence of the mitochondrial DNA control region was obtained from the fecal DNA of 210 rhesus macaque individuals in captivity. A total of 69 haplotypes were defined, 51 of which (73.9%) were newly identified. Of all haplotypes, seven were shared between two zoos, and 62 haplotypes (89.8%) appeared only in a specific zoo, indicating a low rate of animal exchange between Chinese zoos. Moreover, there was a relatively high level of genetic diversity among the rhesus macaques (Hd = 0.0623 ± 0.0009, Pi = 0.979 ± 0.003, *K* = 28.974). Phylogenetic analysis demonstrated that all haplotypes were clearly clustered into two major haplogroups—Clade A (southeastern China) and Clade B (southwestern China)—and each major clade contained several small sub-haplogroups. The haplotypes of rhesus macaques from the same zoo were not clustered together for the most part, but scattered among several subclades on the phylogenetic tree. This indicates that the rhesus macaques in most Chinese zoos may originat from a diverse collection of geographical areas. Our results demonstrate that zoos play an important role in the conservation of the genetic diversity of rhesus macaques, as well as provide useful information on the genetic management of captive rhesus macaques.

## Introduction

Rhesus macaques (*Macaca mulatta*) belong to the old-world monkey family (Cercopithecoidea, Cercopithecinae), and their genetic differentiation with humans is dated back to 25 million years ago (Kumar & Hedges, 1998; Perry et al., 2012). They are distributed in a broad geographical region that extends from Afghanistan in the west to the coast of the East China Sea in the east (Gibbs et al., 2007; Groves, 1985), and over than 60% of provinces in China have populations of rhesus monkeys (Zhang et al., 2016). Morphological and genetic analyses have indicated that the rhesus macaques in China are divided into six subspecies: *M. m. brevicaudus* (Hainan Island, Wanshan Islands), *M. m. tcheliensis* (Northern Henan, Southern Shanxi, Xining, Nanning), *M. m. littoralis* (Fujian, Zhejiang, Anhui, Nanjing, Jiangxi, Northern Guangxi, Guizhou, Eastern Sichuan, Shaanxi, and so on), *M. m. lasiotis* (Northwest Yunnan, western Sichuan, Southeastern Qinghai, Guizhou), *M. m. mulatta* (Yunnan, Southwest Guangxi), and *M. m. vestita* (Tibet) (Jiang, Wang & Ma, 1991; Li et al., 2011; Zhang et al., 2016).

In recent years, rhesus macaque populations have declined in number dramatically due to human-driven deforestation and fragmentation of secondary forests. Therefore, this species has been listed on the CITES Appendix II, as well as defined as a Category II species in the Chinese Wildlife Protection Act (Xu et al., 2010b). Rhesus macaques are raised in almost every Chinese zoo due to their likeability and ease in feeding. There have been some studies on the genetic diversity of captive macaques from the breeding centers of China using molecular markers, such as mitochondrial and microsatellite DNA (Kanthaswamy et al., 2009; Li, 2009; Satkoski et al., 2008; Kanthaswamy & Smith, 2004). However, there have been very few reports on the genetic diversity of Chinese rhesus macaques kept in zoos.

The hypervariable non-coding control region (CR) is a region of mitochondrial DNA (mtDNA) that has the highest incidence of polymorphisms and the fastest evolution (Brown, George & Wilson, 1979; Tamura & Nei, 1993), making it an effective molecular marker in the study of species origin and population genetics (Abdullah et al., 2014; Baxter et al., 2009). In this study, we obtained partial mtDNA CR sequences from 210 rhesus macaques from 11 zoos in China to comprehensively analyze their genetic diversity and phylogenetic relationships. Our results show that zoos play an important role in the conservation of the genetic diversity of rhesus macaques, as well as provide useful information on the genetic management of captive rhesus macaques.

## Materials and Methods

### Ethics statement

The use of all animals and relevant field work were in strict compliance with the Wildlife Protection Law and Nature Reserve Management Regulations of China and was approved by the College of Life Science, Sichuan Agricultural University (Approval number: SKYS20160706).

### Sample collection

**Figure 1:**
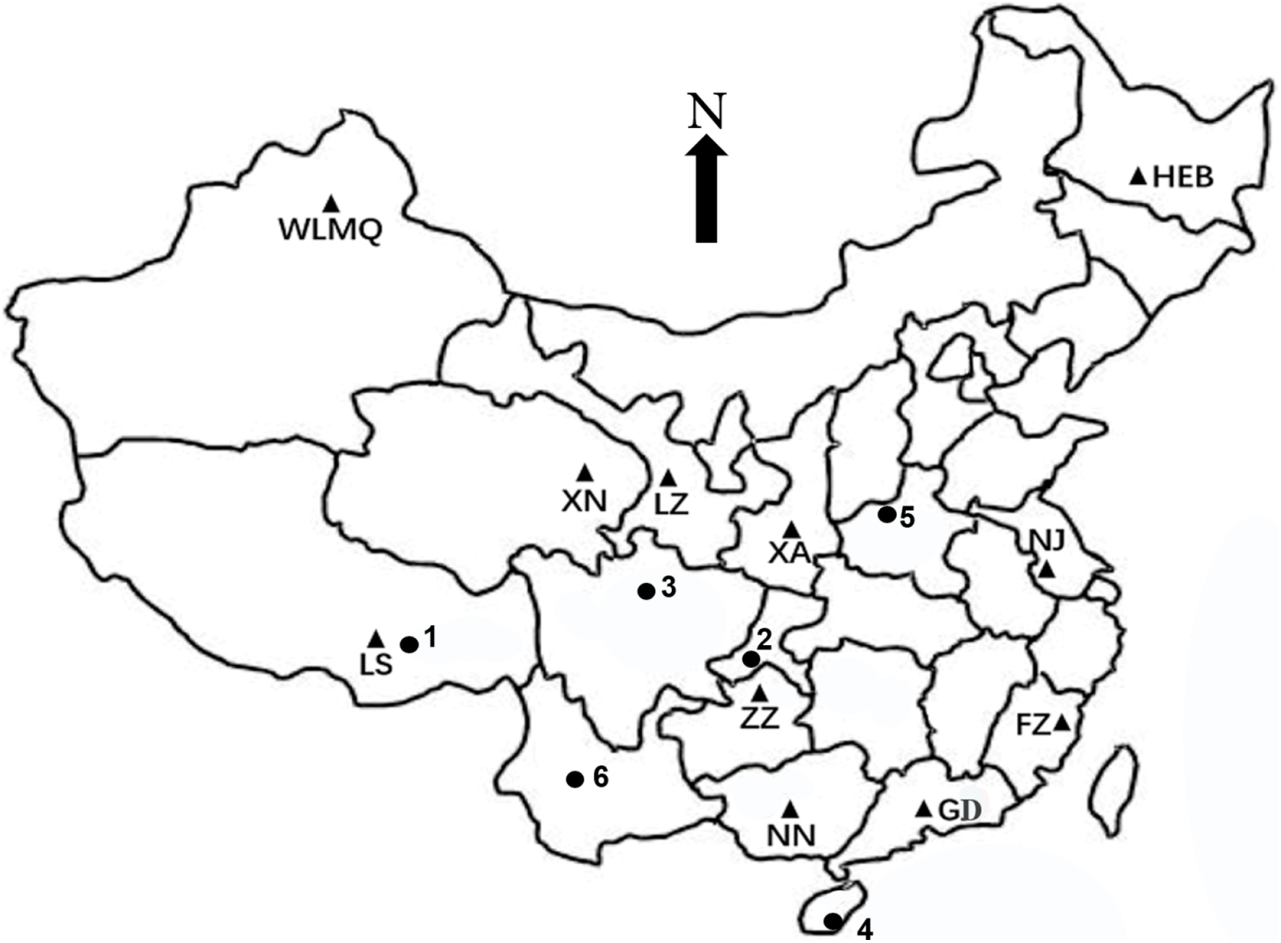
Distribution map of the collection locations of rhesus macaque fecal samples. ▲ represents samples from the zoo populations. Different morphological subspecies barcodes acted by wild individuals: 1—Tibet (*M. m. vestita*), 2—Chongqing (*M. m. littoralis*), 3—Sichuan (*M. m. lasiotis*), 4—Hainan (*M. m. brevicaudus*), 5—Henan (*M. m. tcheliensis*), 6—Yunnan (GenBank accession number: JN863915).

A total of 210 fresh fecal samples were collected non-invasively from captive rhesus macaques located in 11 zoos at different geographical locations in China (Table 1). Five wild fecal samples were collected from Hainan, Henan, Chongqing, Sichuan, and Tibet, representing five subspecies of rhesus macaques: *M. m. brevicaudus, M. m. tcheliensis, M. m. littoralis, M. m. lasiotis, and M. m. vestita*, respectively. Corresponding sequences for each of these subspecies was obtained from NCBI (GenBank accession number: AF135317, AF135308, AF135303, GQ472261, NC037466). Due to sampling difficulty, five sequences of the subspecies *M. m. mulatta* from Yunnan were obtained from NCBI (GenBank accession number: JN863915, AY646971, AY646974, AY646979, and AF135352). The specific sources of all samples are described in Fig. 1. During sample collection, the whole fresh feces of each individual was put into an aseptic bag, transported to the laboratory using a sample collection kit on dry ice, and stored at −80 °C until use.

**Table 1:** The sample information and the genetic diversity parameters of different *M. mulatta* populations in 11 Chinese zoos.

Populations	Sample size	Number of variable sites, Vs	Number of haplotypes, *H*	Nucleotide diversity (per site), Pi	Haplotype diversity, Hd	Average number of pairwise nucleotide differences, *K*
ZZ	38	80	15	0.0483 ± 0.0040	0.915 ± 0.024	22.767
FZ	13	38	3	0.0385 ± 0.0055	0.603 ± 0.088	18.179
LZ	19	66	4	0.0558 ± 0.0035	0.766 ± 0.049	27.906
NJ	14	56	9	0.0465 ± 0.0054	0.912 ± 0.059	22.088
XA	7	67	6	0.0599 ± 0.0077	0.952 ± 0.096	29.429
XN	38	76	7	0.0545 ± 0.0028	0.844 ± 0.025	25.822
HEB	24	82	10	0.0509 ± 0.0059	0.902 ± 0.031	23.928
LS	17	2	2	0.0019 ± 0.0004	0.441 ± 0.098	0.882
NN	9	32	4	0.0232 ± 0.0071	0.806 ± 0.089	11.000
GD	12	43	5	0.0256 ± 0.0069	0.667 ± 0.141	12.167
WLMQ	19	41	11	0.0307 ± 0.0037	0.942 ± 0.030	14.526
Total	210	124	69	0.0623 ± 0.0009	0.979 ± 0.003	28.974

**Note:**

ZZ represent Zhazuo zoo, Guizhou; FZ represent Fuzhou zoo, Fujian; LZ represent Lanzhou zoo, Gansu; NJ represent Nanjing Hongshan zoo, Jiangsu; XA represent Qinling zoo, Shanxi; XN represent Xining zoo, Qinghai; HEB represent Harbin zoo, Heilongjiang; LS represent Norbulingka zoo, Tibet; NN represent Nanning zoo, Guangxi; GD represent Guangzhou zoo, Guangdong; WLMQ represent Tianshanwildzoo, Xinjiang.

### DNA extraction and mtDNA amplification and sequencing

About 1.5–2.5 g of the fecal epidermis was collected by scraping with a sterile toothpick and used to extract DNA. Total genome DNA was extracted using a QIAamp DNA Stool kit (Qiagen, Hilden, Germany) according to the manufacturer’s instructions. Primer Premier 5.0 software and the complete mtDNA sequence (NC005943) of *M. mulatta* (Gokey et al., 2004) was used to design the following pair of primers to amplify a 475 base pair (bp) CR fragment: forward primer: 5′-TCC GAG GGC AAT CAG AAA GAA A-3′, reverse Primer: 5′-GCC TTG AGG TAA GAA CCA GAT GC-3′ (synthesized by TSINGKE (Chengdu) Biotechnology Co., Ltd, People’s Republic of China). Polymerase chain reaction (PCR) was carried out in a reaction volume of 20 μL containing 40 ng of DNA template, one pmol of each primer, 0.4 mM of each dNTP, three mM MgCl_2_, and one U of Taq polymerase (Medigen, Novosibirsk, Russia). The PCR procedure used was as follows: initial denaturation for 5 min at 95 °C, 35 cycles of 95 °C for 50 s, annealing at 55 °C for 45 s, and primer extension at 72 °C for 60 s. A final extension was carried out at 72 °C for 10 min. PCR products were detected by gel electrophoresis using a 1% agarose gel and then bidirectionally sequenced by TSINGKE (Chengdu) Biotechnology Co., Ltd.

### Data analysis

Sequences were edited and aligned using SeqMan DNAstar Software (Siasi et al., 2012). *Sequence homologies* were compiled and compared using MEGA7.0 software (Kumar, Stecher & Tamura, 2016). DnaSP5.0 software was used to calculate the number of haplotypes (*H*), haplotype diversity (Hd), nucleotide diversity (Pi), and average number of nucleotide differences (*K*). Base frequency and compositional bias were analyzed in PAUP* 4.0 (Wilgenbusch & Swofford, 2003). Phylogenetic analysis was performed using the MrBayes3.1.2 (Ronquist & Huelsenbeck, 2003) and PAUP* 4.0 software packages. Bayesian inference (BI), maximal-likelihood (ML), neighbor-joining distance (NJ), and maximum-parsimony (MP) methods were used to construct a phylogenetic tree in MrBayes3.1.2.

**Figure 2:**
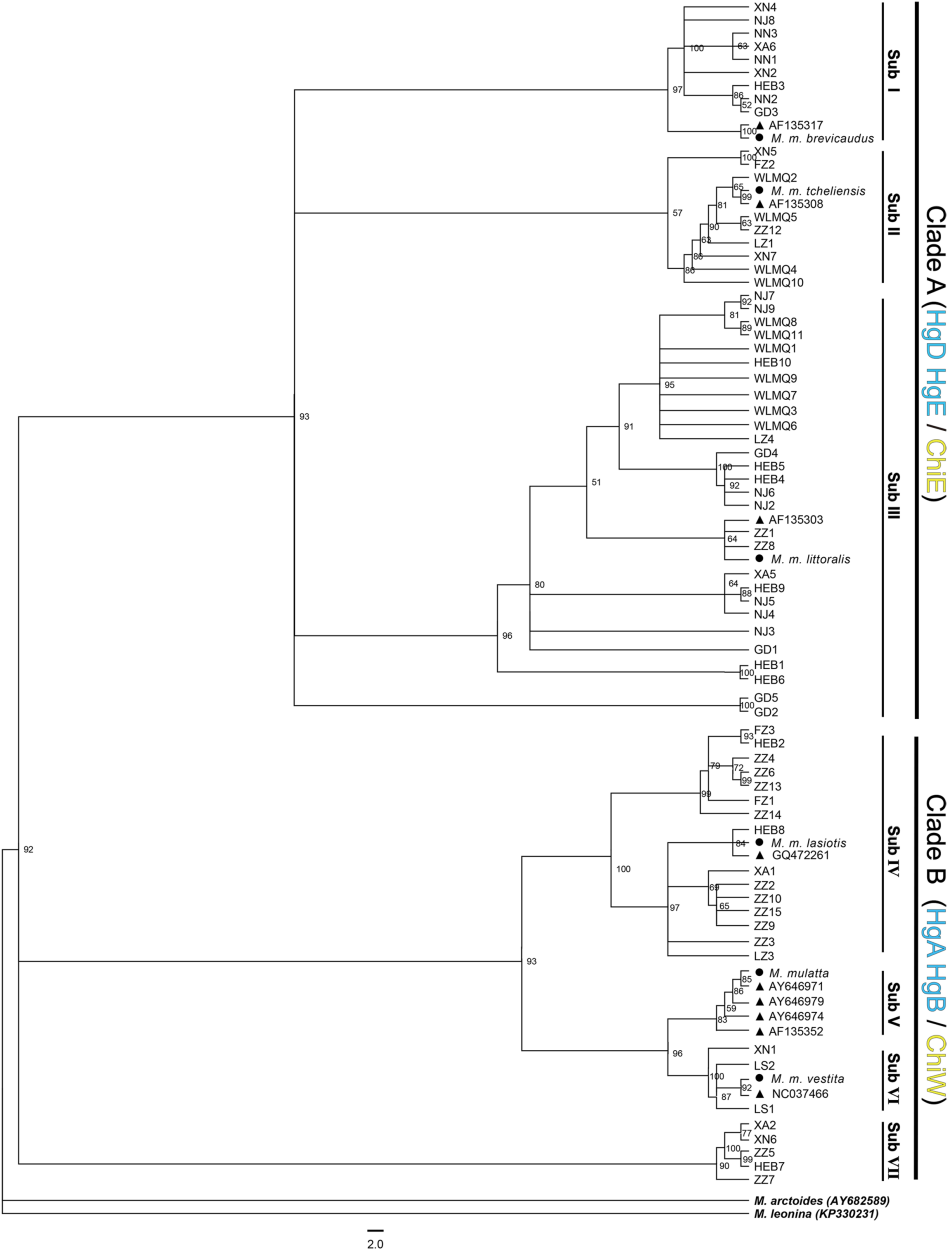
Bayesian phylogenetic tree constructed based on 69 captive mtDNA haplotypes and 15 corresponding wild-origin sequences. ● represents the Chinese subspecies based on five wild sequences except *M. mulatta*, which is represented by one of the Yunnan sequences from NCBI. ▲ represents the other nine wild sequences obtained from NCBI, which originated from Hainan Island, Henan, Guangxi, Sichuan, Yunnan, and Tibet. The northern pig-tailed macaque *(M. leonina*) and the stump-tailed macaque (*M. arctoides*) were used as outgroups. Numbers at tree nodes are assigned probabilities, and haplogroup designations are identified by vertical bars at the right. Sub I–VII represents subclades I–VII. The blue labels, HgA, HgB, HgD, and HgE, represent haplogroups A, B, D, and E as defined by Li et al. (2011). The yellow labels, ChiE and ChiW, represent haplogroups defined by Smith & McDonough (2005). ZZ1∼15, FZ1∼2, LZ1∼5, NJ1∼11, XA1∼6, XN1∼7, HEB1∼10, LS1∼2, NN1∼4, GD1∼5, WLMQ1∼11 represent haplotypes from ZZ, FZ, LZ, NJ, XA, XN, HEB, LS, NN, GD, and WLMQ zoo, respectively.

The evolutionary model was analyzed with jModeltest version 0.1.1 (Posada, 2008). The best fitting evolutionary model was the HKY+I+G model (Hasegawa, Kishino & Yao, 1985), with a proportion of invariable sites and rate variation. Four MCMC chains were run for 100,000 generations at the same time, with sampling every 1,000 generations. The first 250 aging samples and the last 250 aging samples were discarded to get tree files. Values from a replicate bootstrap analysis were added to the branches of the trees. In ML, NJ, and MP analyses, heuristic searches and tree-bisection reconnection were selected for tree construction, and non-parametric bootstrap support values were obtained by resampling and analyzing 250, 1,000, and 1,000 replicates (Felsenstein, 1985). The NJ tree was constructed from the F84 distance matrix without assuming a molecular clock. MP analysis was scored by the maximum-likelihood criterion. All trees were edited by Figtree (Rambaut, 2012). The northern pig-tailed macaque (*M. leonina*) and the stump-tailed macaque (*M. arctoides*) were used as outgroups of the phylogenetic tree (Fig. 2). Network 4.611 (Hasan et al., 2015) was used to construct an unrooted median-joining (MJ) haplotype network diagram.

## Results

### Sequence characteristics of mtDNA in 11 zoo monkey populations

The 475-bp mtDNA CR fragment isolated from 210 Chinese rhesus macaques was sequenced. After alignment, 69 haplotypes were defined (GenBank accession numbers: MH730669–MH730749; Data S1), 51 of which were novel to this study. Of all haplotypes, seven were shared between two zoos, and 62 haplotypes (89.8%) appeared only in a specific zoo. The distribution of haplotypes in each population are shown in Table 1. A total of 351 conserved sites (Cs) and 124 variable sites (Vs) were detected, including 12 singleton sites and 112 parsimony informative sites, accounting for 26.1% of the total analysis sites. These Vs included 93 transitions, 30 transversions and one indel. The base composition of the mtDNA CR revealed that the sequences are apparently AT biased (AT = 61.0%), with an overall base composition of 31.9% A, 11.0% G, 29.1% T, and 28.0% C.

### Genetic diversity of the 11 rhesus macaque populations

To explore the genetic diversity between the macaques at the 11 zoos, we calculated the Hd, Pi, and the average number of nucleotide difference (*K*) for both the entire studied population and each zoo population (Table 1). The overall Pi, Hd, and *K* values were 0.0623 ± 0.0009, 0.979 ± 0.003, and 28.974, respectively, which demonstrates that there is a high level of genetic diversity in Chinese zoos. Among the 11 captive populations, the highest value of Pi was found in the XA zoo (0.0599), followed by the LZ zoo (0.0558), and the lowest was found in the LS zoo (0.0019). The highest Hd value (0.952) was found in the XA zoo, followed by the WLMQ zoo (0.942), and the lowest Hd value (0.441) was found in the LS zoo.

### Phylogenetic analysis

Phylogenetic trees were constructed using BI, NJ, ML, and MP methods based on the 69 captive mtDNA haplotypes and 15 corresponding wild-origin sequences (Fig. 2; Figs. S1–S3). All trees exhibited similar topological structures, and there was a high bootstrap value for each major clade. The BI tree was used as an example in this study (Fig. 2). The BI tree showed that all haplotypes clearly clustered into two major haplogroups (Clade A and B) with a relatively high bootstrap value (92% support), and each major clade consisted of several, small sub-haplogroups. Clade A included three subclades (Subs I–III), and Clade B included four subclades (Subs IV–VII). A total of 15 wild-origin sequences representing six subspecies were clustered and scattered in Subs I–VI, and Sub VII was composed of only captive haplotypes.

The BI showed that, for most zoos, the haplotypes of the rhesus macaques from the same zoo were not clustered together, but scattered into several subclades on the phylogenetic tree. This indicates that the rhesus macaques in most Chinese zoos may have been originally captured from a diverse set of geographical regions. For example, the 10 haplotypes from the HEB zoo were scattered into four subclades (Subs I, III, IV, and VII), while all haplotypes from the LS zoo were clustered together to form one subclade (Sub VI). However, for most subclades, each subclade was composed of haplotypes from different zoos.

**Figure 3:**
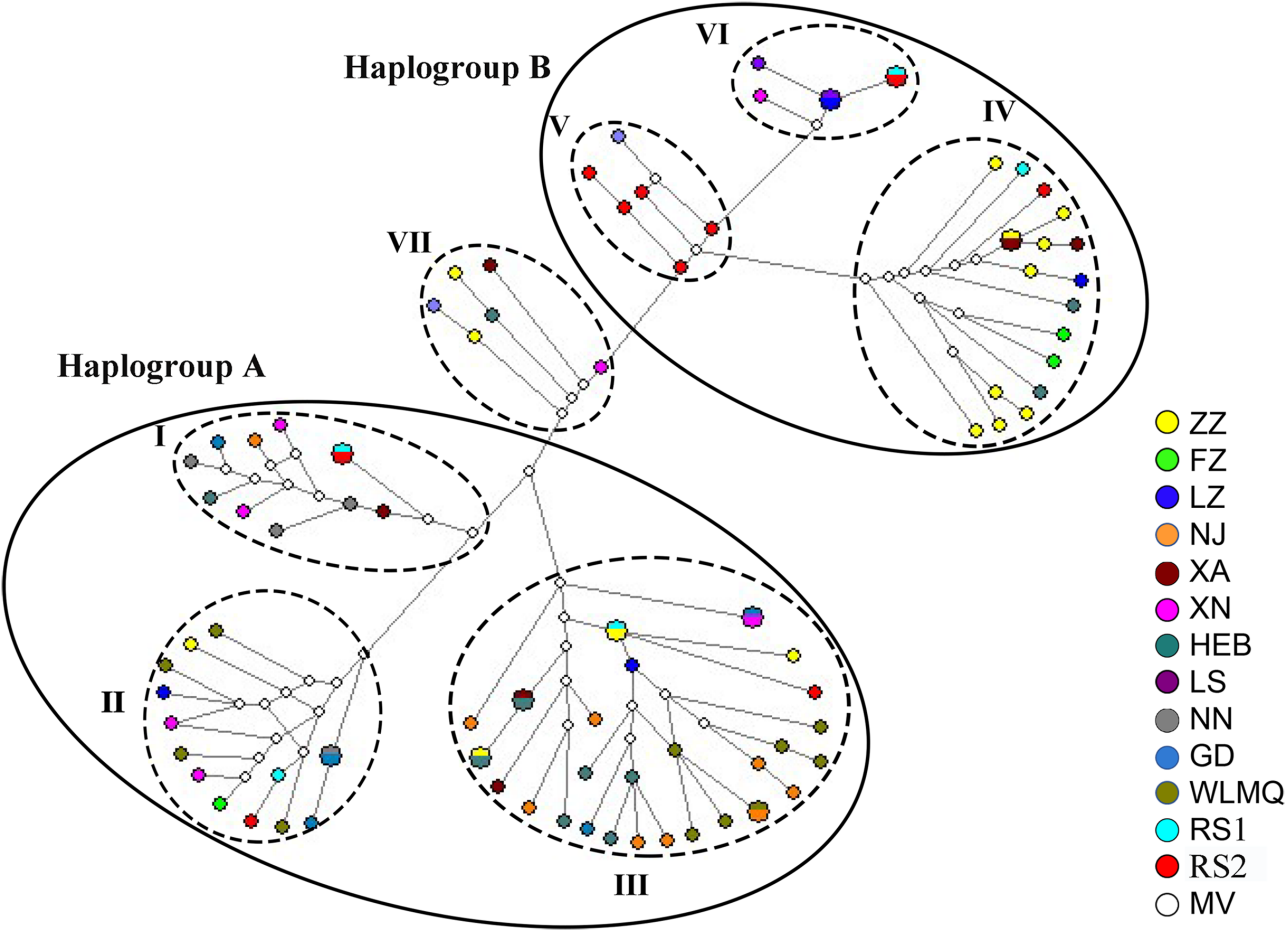
Median-joining haplotype network for 69 zoo haplotypes and 15 corresponding wild-origin sequences. RS1 represents the wild reference sequences of *M. m. brevicaudus, M. m. tcheliensis, M. m. littoralis, M. m. lasiotis*, and *M. m. vestita* subspecies, from which the five wild fecal samples used in the experiments were collected. RS2 represents the wild reference sequences obtained from NCBI, including one corresponding sequence for each subspecies, except *M. m. mulatta*, which included five wild sequences from Yunnan. MV represents the median vector of these haplotypes. The size of the circles represents the number of samples for each specific haplotype.

In addition, the MJ haplotype network diagram (Fig. 3) clearly clustered seven sub-haplogroups (I–VII) into two distinct haplogroups: Haplogroups A and B. The three sub-haplogroups identified in eastern China (Subs I–III) formed a unique haplogroup, Haplogroup A, while the four sub-haplogroups identified in western China (Subs IV–VI) formed another haplogroup, Haplogroup B. Sub-haplogroup VII consisted of only five zoo haplotypes (XA2, XN6, ZZ5, HEB7, and ZZ7) and formed a single sub-haplogroup located between the haplogroups A and B.

## Discussion

Through sequence alignment, 69 distinct haplotypes of rhesus macaques were identified among captive populations in zoos, seven of which were shared between two zoos. The remaining 62 haplotypes (89.8%) only appeared in a specific zoo and were not shared between zoos (Table S1), suggesting a low rate of animal exchange between Chinese zoos. Moreover, 51 novel haplotypes (73.9%) were detected for the first time in this study, indicating that zoos may play an important role in the conservation of genetic diversity in Chinese rhesus macaques.

Genetic diversity is typically measured by Hd and Pi, both of which reflect the magnitude of genetic variation at different scales (Tajima, 1983; Nei, 1987). The values of Hd and Pi were estimated for both the whole population (all 210 macaque individuals) and each zoo population, and the values for the whole population were higher (Hd = 0.979, Pi = 0.0623) than those measured in the individual zoo populations, demonstrating a high level of genetic diversity between the zoo populations. Previous studies have reported the genetic diversity of wild Chinese rhesus macaques in Yunnan (Pi = 0.020, Hd = 0.955) (Yu et al., 2012), Sichuan (Pi = 0.0148, Hd = 0.686) (Xu et al., 2010a), Taihangshan, Henan (Pi = 0.0106, Hd = 0.872) (Xie, Lu & Wang, 2011), and Nanwan, Hainan (Hd = 0.0026) (Tao, 2009). The genetic diversity between the populations of the 11 zoos in this study was high compared to these wild populations, likely due to the greater heterogeneity of geographic origin of the animals at Chinese zoos (Satkoski et al., 2008). Of the 11 investigated zoos, all zoos but one have high Hd > 0.5 and Pi > 0.02. The LS zoo has the lowest Hd = 0.441 and Pi = 0.0019, which may be explained by the fact that the LS zoo has not introduced new macaque individuals since its establishment in 1768.

Phylogenetic analysis showed that all haplotypes clearly clustered into two major haplogroups (Clades A and B), and that each major clade consisted of several small sub-haplogroups (Subs I–VII). A total of 15 wild-origin sequences representing six subspecies were clustered into Subs I–VI, indicating a clear maternal geographical origin of the six subclades. Subs I–III of Clade A corresponded to the subspecies *M. m. brevicaudus*, *M. m. tcheliensis*, and *M. m. littoralis*, respectively, and were mainly distributed in southeastern China. Subs IV–VI of Clade B corresponded to the subspecies *M. m. lasiotis*, *M. m. mulatta*, and *M. m. vestita*, respectively, and were mainly distributed in southwestern China. The MJ haplotype network (Fig. 3) had a structure similar to that of the tree. This implies that there may be two major maternal origins of rhesus macaques in southeastern and southwestern China. These results are consistent with those reported in previous studies based on wild rhesus macaque samples (Wu et al., 2013; Li et al., 2011; Smith & McDonough, 2005). Clades A and B identified in this study corresponded to haplogroup ChiE (eastern) and ChiW (western), respectively, categories defined by Smith & McDonough (2005). Clades A and B also corresponded to haplogroup D–E and haplogroup A–B, respectively, which were defined by Li et al. (2011). Sub VII formed a separate sub-haplogroup and did not contain any representative subspecies haplotypes, indicating that it may represent a new subspecies.

Zoos typically receive animals from nearby areas, suggesting that haplotypes within the same zoo should be clustered together. However, we show, in this study, that the haplotypes of rhesus macaques from the same zoo, in most cases, were not clustered together, but scattered into several subclades on the phylogenetic tree. These results indicate that the rhesus macaques in most Chinese zoos may originally be derived from more diverse geographical regions and not just the areas nearby the zoos.

## Conclusions

The high Hd and Pi found in the whole population indicates a high genetic diversity among the zoos, except for the LS zoo, which has the lowest Hd and Pi values. Moreover, there may be two major maternal origins of captive Chinese rhesus macaques—one in southeastern China and one in southwestern China. Rhesus macaques in Chinese zoos have diverse, heterogenic geographic origins. Taken together, these data suggest that Chinese zoos may play an important role in the conservation of genetic diversity of Chinese rhesus macaques.

This study provides insight into the genetic management and conservation of captive Chinese rhesus macaques. When new rhesus macaque individuals are introduced into zoos, their geographical origin and genetic background should be taken into account, and more living spaces should be made available to fertile females to avoid inbreeding. *In addition*, when the genetic diversity of zoo populations has been lost, both stabilizing population size and maintaining a proper proportion of females to males have been shown to be effective at mitigating the total loss of genetic diversity.

## Supplemental Information

10.7717/peerj.6957/supp-1 Supplemental Information 1 Haplotype sequence information of rhesus macaques. A total of 74 haplotype sequences, including 69 haplotypes from 11 Chinese zoos and 5 haplotypes from the wild. Haplotype name and GenBank accession number were shown in bold type before each sequence. For example, in the “**ZZ1 MH730725**,” “**ZZ1**” is haplotype name, and “**MH730725**” is GenBank accession number. The number in the upper right corner of haplotype names represent the shared haplotype between two zoos.

10.7717/peerj.6957/supp-2 Supplemental Information 2 Maximal likelihood phylogenetic tree constructed based on the 69 captive mtDNA haplotypes and 15 corresponding wild-origin sequences. ● represents the Chinese subspecies based on five wild sequences except *M. mulatta*, which is represented by one of the Yunnan sequences from NCBI. ▲ represents the other nine wild sequences obtained from NCBI, which originated from Hainan Island, Henan, Guangxi, Sichuan, Yunnan, and Tibet. The northern pig-tailed macaque *(M. leonina*) and the stump-tailed macaque (*M. arctoides*) were used as outgroups. Numbers at tree nodes are assigned probabilities, and haplogroup designations are identified by vertical bars at the right. Sub I–VII represents subclades I–VII. The blue labels, HgA, HgB, HgD, and HgE, represent haplogroups A, B, D, and E as defined by Li et al. (2011). The yellow labels, ChiE and ChiW, represent haplogroups defined by Smith & McDonough (2005). ZZ1∼15, FZ1∼2, LZ1∼5, NJ1∼11, XA1∼6, XN1∼7, HEB1∼10, LS1∼2, NN1∼4, GD1∼5, WLMQ1∼11 represent haplotypes from ZZ, FZ, LZ, NJ, XA, XN, HEB, LS, NN, GD and WLMQ zoo, respectively.

10.7717/peerj.6957/supp-3 Supplemental Information 3 Maximum parsimony phylogenetic tree constructed based on 69 captive mtDNA haplotypes and 15 corresponding wild-origin sequences. ● represents the Chinese subspecies based on five wild sequences except *M. mulatta*, which is represented by one of the Yunnan sequences from NCBI. ▲ represents the other nine wild sequences obtained from NCBI, which originated from Hainan Island, Henan, Guangxi, Sichuan, Yunnan, and Tibet. The northern pig-tailed macaque *(M. leonina*) and the stump-tailed macaque (*M. arctoides*) were used as outgroups. Numbers at tree nodes are assigned probabilities, and haplogroup designations are identified by vertical bars at the right. Sub I–VII represents subclades I–VII. The blue labels, HgA, HgB, HgD, and HgE, represent haplogroups A, B, D, and E as defined by Li et al. (2011). The yellow labels, ChiE and ChiW, represent haplogroups defined by Smith & McDonough (2005). ZZ1∼15, FZ1∼2, LZ1∼5, NJ1∼11, XA1∼6, XN1∼7, HEB1∼10, LS1∼2, NN1∼4, GD1∼5, WLMQ1∼11 represent haplotypes from ZZ, FZ, LZ, NJ, XA, XN, HEB, LS, NN, GD and WLMQ zoo, respectively.

10.7717/peerj.6957/supp-4 Supplemental Information 4 Neighbor-joining phylogenetic tree constructed based on 69 captive mtDNA haplotypes and 15 corresponding wild-origin sequences. ● represents the Chinese subspecies based on five wild sequences except *M. mulatta*, which is represented by one of the Yunnan sequences from NCBI. ▲ represents the other nine wild sequences obtained from NCBI, which originated from Hainan Island, Henan, Guangxi, Sichuan, Yunnan, and Tibet. The northern pig-tailed macaque *(M. leonina*) and the stump-tailed macaque (*M. arctoides*) were used as outgroups. Numbers at tree nodes are assigned probabilities, and haplogroup designations are identified by vertical bars at the right. Sub I–VII represents subclades I–VII. The blue labels, HgA, HgB, HgD, and HgE, represent haplogroups A, B, D, and E as defined by Li et al. (2011). The yellow labels, ChiE and ChiW, represent haplogroups defined by Smith & McDonough (2005). ZZ1∼15, FZ1∼2, LZ1∼5, NJ1∼11, XA1∼6, XN1∼7, HEB1∼10, LS1∼2, NN1∼4, GD1∼5, WLMQ1∼11 represent haplotypes from ZZ, FZ, LZ, NJ, XA, XN, HEB, LS, NN, GD and WLMQ zoo, respectively.

10.7717/peerj.6957/supp-5 Supplemental Information 5 Distribution of 69 haplotypes in 11 zoo rhesus macaque populations in China. The parenthesized numbers are sample quantities and the underlined represent shared haplotypes between zoos. **^1∼7^** represent haplotypes shared by zoos.

## Additional Information and Declarations

### Field Study Permissions

The following information was supplied relating to field study approvals (i.e., approving body and any reference numbers): All field work was approved by the College of Life Science, Sichuan Agricultural University (Approval number: SKYS20160706).

### Data Availability

The following information was supplied regarding data availability: Data is available at NCBI GenBank, accession numbers: MH730669–MH730749.
