# Supplementary material for: Genetic diversity and phylogenetic analyses of 11 cohorts of captive rhesus macaques from Chinese zoos
Source: PeerJ. 2019 May 29;7:e6957. doi: 10.7717/peerj.6957 (PMC6545102; doi:10.7717/peerj.6957)
Supplement: Supplemental Information 1 — A total of 74 haplotype sequences, including 69 haplotypes from 11 Chinese zoos and 5 haplotypes from the wild. Haplotype name and GenBank accession number were shown in bold type before each sequence. For example, in the “ZZ1 MH730725,” “ZZ1” is haplotype name, and “MH730725” is GenBank accession number. The number in the upper right corner of haplotype names represent the shared haplotype between two zoos. [file peerj-07-6957-s001.docx]

**Haplotype sequence information of rhesus macaques**

A total of 74 haplotype sequences, including 69 haplotypes from 11 Chinese zoos and 5 haplotypes from the wild. Haplotype name and GenBank accession number were shown in bold type before each sequence. For example, in the “**ZZ1 MH730725**”, “**ZZ1**” is haplotype name, and “**MH730725**” is GenBank accession number. The number in the upper right corner of haplotype names represent the shared haplotype between two zoos.

**ZZ1 MH730725**

GGCATTCTAACTTAAACTACTTTCTGCATTCTTATGTTGTATAAACTTCATAAAATAACCCTGACACCAACCTACTCATAATACTGCTATGTAATTCGTGCATTACTGCTAGTCAGCATGTATAATATATAGTACTATATATGTTTAACTGTACATAACACATATTATTACATATCAACTTAACATTCTAAACAACATGCTTACAAGCAAGTACCTTAATACAGACCTCAACAGTAGTACATAGCATGATTCCTTCAAATTCAACTTGTCCTCCTCATGAGTATCAACTAAACCAGTCCATGCCAGTCGTCCATAGTACATTAAATCGTTCATCGGACATAGCACATATTCATTAAATAATCCTCCTCACCACGGATGCCCCCCCTCACTTAGGAGTCCCTTGTTCACCATCCTCCGTGAAATCAATATCCCGCACAAGAGTGCTACTCTCCTCGCTCCGGGCCCATAACTCGTGGGGGTAGCTATACTTGAGCTGTATCCG

**ZZ2^1^  MH730742**

GGCATTCTAACTTAAACTACTTTCTGTATTCTTACGTTGCACAACTTCATAAAATAACCCTAACATTCAACCTACCCATAATATTACTATGTAATTCGTGCATTACTGCTAGCCAACATGTATAATATATAGTACTATATATGCTTAACTGTACATAACACATATCATTACATATCAACTCAACATCCTAGACAACATGCTTACAAGCAAGTACTCTAATACAAGCCCCAACAGTAGCACATAATATGGCCTCTCCAAATTCAATTCACCTCCTCATGAATACCAACCGAACCAGTCCATGCCAGTCGTCCATAGTACATTAAATCGTTCATCGGACATAGCACATATTCATTAAATAATCCTCCTCACCACGGATGCCCCCCCTCACTTAGGAATCCCTTGTTCACCATCCTCCGTGAAATCAATATCCCGCACAAGAGTGCTACTCTCCTCGCTCCGGGCCCATAACTCGTGGGGGTAGCTATACTCGAGCTGTATCCG

**ZZ3 MH730736**

GGCATTCTAACTTAAACTACTTTCTGTATTCTTATGTTGCACAACTTCATAAAATAACCCTAACATTCAACCTACCCATAATATTACTATGTAATTCGTGCATTACTGCTAGCCAACATGTATAATATATAGTACTATATATGCTTAACTGTACATAACACATATCATTACATATCAACTCAACATCCTAGACAACATGCTTACAAGCAAGTACTCTAATACGAGTTCCAACAGTAGTACATAATATGGCCCCTCCAAATTCAATCCACTTCCTCATGAATACCAACCGAACCAGTCCATGCCAATCGTCCATAGTACATTAAATCGTTCATCGGACATAGCACATATTCATTAAATAATCCTCCTCACCACGGATGCCCCCCCTCACTTAGGAGTCCCTTGTTCACCATCCTCCGTGAAATCAATATCCCGCACAAGAGTGCTACTCTCCTCGCTCCGGGCCCATAACTCGTGGGGGTAGCTATACTTGAGCTGTATCCG

**ZZ4 MH730676**

GGCATTCTAACTTAAACTACTTTCTGTATTCTTATGTTGCATAACTTCATAAAATAACCCTAACATCAACCCACCCATAATATTACTATGTAATTCGTGCATTACTGCTAGCCAACATGTATAATATATAGTACTATATATGCTTAACTGTACATAACACATATCATTACATATCAACCCAACATTCCAGACAACATGCTTACAAGCAAGTACTCTAATACAAGTCCCAACAGTAATACATAGTATGGCCCTTCCAAATTCAATCCGTCCCCCCCCATGGATATCAACCAAACCAGTCCATGCCAGTCGTCCATAGTACATCAAATTGTTCATCGGACATAGTACATATTCATTAAATAATCCTCCTCACCACGGATGCCCCCCCTCACTTAGGAGTCCCTTGTTCACCATCCTCCGTGAAATCAATATCCCGCACAAGAGTGCTACTCTCCTCGCTCCGGGCCCATAACTCGTGGGGGTAGCTATACTTGAGCTGTATCCG

**ZZ5 MH730674**

GGCATTCTAACTTAAACTACTTTCTGCATTCTTATGTTGTATAAACTTCATAAAACAACCCTAACACCAACCTACTCATAATACTACTATGTAATTCGTGCATTACTGCTAGCCAACATGTATAATATATAGTACTATATATGCTTAACTGTACATAACACATACCATTACATATCAACTTAACATTCTAGACAACATGCTTACAAGCAAGTACTCTAATATAAACTTCAGCAGTAACACATAATATGGCTTCTCCAAATTTGACCTGTCCTCCTCATGAATATCGACCAAACCAGTTCATGCCAGTCGTCCATAGTACATTAAATCGTTCATCGGACATAGCACATATTCATTAAATAATCCTCCTCACCACGGATGCCCCCCCTCACTTAGGAATCCCTTATTCACCATCCTCCGTGAAATCAATATCCCGCACAAGAGTGCTACTCTCCTCGCTCCGGGCCCATAACTCGTGGGGGTAGCTATACTTGAGCTGTATCCG

**ZZ6 MH730678**

GGCATTCTAACTTAAACTACTTTCTGTATTCTTATGTTGCATAACTTCATAAAATAACCCTAACATCAACCCACCCATAATATTACTATGTAATTCGTGCATTACTGCTAGCCAACATGTATAATATATAGTACTATATATGCTTAACTGTACATAACACATATCATTACATATCAACCCAACATTCCAGACAACATGCTTACAAGCAAGTACTCTAATACAAGTCCCAACAGTAATACATAGTATGGCCCTTCCAAATTCAATCTGTCCCCCCTCATGGATATCAACCAAACCAGTCCATGCCAGTCGTCCATAGTACATTAAATTGTTCATCGGACATAGTACATATTCATTAAATAATCCTCCTCACCACGGATGCCCCCCCTCACTTAGGAGTCCCTTGTTCACCATCCTCCGTGAAATCAATATCCCGCACAAGAGTGCTACTCTCCTCGCTCCGGGCCCATAACTCGTGGGGGTAGCTATACTTGAGCTGTATCCG

**ZZ7 MH730696**

GGCATTCTAACTTAAACTACTTTCTGCATTCTTATGTTGTATAAACTTCATAAAACAACCCTAACACCAACCTACTCATAATACTACTATGTAATTCGTGCATTACTGCTAGCCAGCATGTATAATATATAGTACTATATATGCTTAACTGTACATAACACATATTATTACATATCAACTTAACATTCTAGACAACATGCTTACAAGCAAGCACTCTAATACAAACTTCAACAGTAACACATAATATGGCTTCTCCAAATTTGACCTATCTTCCTCATGAATATCAACCGAACCAGTCCATGCCAGTCGTCCATAGTACATTAAATCGTTCATCGGACATAGCACATATTCATTAAATAATCCTCCTCACCACGGATGCCCCCCCTCACTTAGGAATCCCTTGTTCACCATCCTCCGTGAAATCAATATCCCGCACAAGAGTGCTACTCTCCTCGCTCCGGGCCCATAACTCGTGGGGGTAGCTATACTTGAGCTGTATCCG

**ZZ8 MH730727**

GGCATTCTAACTTAAACTACTTTCTGCATTCTTATGTTGTATAAACTTCATAAAATAACCCTGACACCAACCTACTCATAATACTGCTATGTAATTCGTGCATTACTGCTAGTCAGCATGTATAATATATAGTACTATATATGTTTAACTGTACATAACACATATTATTACATATCAACTTAACATTCTAAACAACATGCTTACAAGCAAGTACCTTAATACAAACCTCAACAGTAGTACATAGCATGATTCCTTCAAATTCAACTTGTCCTCCTCATGAGTATCAACTAAACCAGTCCATGCCAGTCGTCCATAGTACATTAAATCGTTCATCGGACATAGCACATATTCATTAAATAATCCTCCTCACCACGGATGCCCCCCCTCACTTAGGAGTCCCTTGTTCACCATCCTCCGTGAAATCAATATCCCGCACAAGAGTGCTACTCTCCTCGCTCCGGGCCCATAACTCGTGGGGGTAGCTATACTTGAGCTGTATCCG

**ZZ9 MH730737**

GGCATTCTAACTTAAACTACTTTCTGTATTCTTACGTTGCACAACTTCATAAAATAACCCTAACATTCAACCTACCCATAATATTACTATGTAATTCGTGCATTACTGCTAGCCAACATGTATAATATATAGTACTATATATGCTTAACTGTACATAACACATATCATTACATATCAACTCAACATCCTAGACAACATGCTTACAAGCAAGTACTCTAATACAAGCTCCAACAGTAGCACATAATATGGCCTCTCCAAATTCAATTCACCTCCCCATGAATACCAACCGAACCAGTCCATGCCAATCGTCCATAGTACATTAAATCGTTCATCGGACATAGCACATATTCATTAAATAATCCTCCTCACCACGGATGCCCCCCCTCACTTAGGAATCCCTTGTTCACCATCCTCCGTGAAATCAATATCCCGCACAAGAGTGCTACTCTCCTCGCTCCGGGCCCATAACTCGTGGGGGTAGCTATACTCGAGCTGTATCCG

**ZZ10 MH730740**

GGCATTCTAACTTAAACTACTTTCTGTATTCTTACGTTGCACAACTTCATAAAATAACCCTAACATTCAACCTACCCATAATATTACTATGTAATTCGTGCATTACTGCTAGCCAACATGTATAATATATAGTACTATATATGCTTAACTGTACATAACACATATCATTACATATCAACTCAACATCCTAGACAACATGCTTACAAGCAAGTACTCTAATACAAGCCCCAACAGTAGCACATAACATGGCCTCTCCAAATTCAATTCACCTCCTCATGAATACCAACCGAACCAGTCCATGCCAGTCGTCCATAGTACATTAAATCGTTCATCGGACATAGCACATATTCATTAAATAATCCTCCTCACCACGGATGCCCCCCCTCACTTAGGAATCCCTTGTTCACCATCCTCCGTGAAATCAATATCCCGCACAAGAGTGCTACTCTCCTCGCTCCGGGCCCATAACTCGTGGGGGTAGCTATACTCGAGCTGTATCCG

**ZZ11^5^ MH730702**

GGCATTCTAACTTAAACTACTTTCTGCATTCTTATGTTGTATAAACTTCATAAAATAACCCTGACACCAACCTACTCATAATACTGCTATGTAATTCGTGCATTACTGCTAGCCAACATGTATAATATATAGTACTATATATGCTTAACTGTACATAACACATATCATTACATATCAACTTAACATTCTAAACAACATGCTTACAAGCAAGTACTTTAATACGAACCTCAACAGTAGTACATAACATGATTTCTTCAAATTCAACTTGTCCCTCCCCATGAGTATCAACTAGACCAACTCATGCCAGTCGTCCATAGTACATTAAATCGTTCATCGGACATAGCACATATTCATTAAATAATCCTCCTCACTACGGATGCCCCCCCTCACTTAGGAGTCCCTTGTTCACCATCCTCCGTGAAATCAATATCCCGCACAAGAGTGCTACTCTCCTCGCTCCGGGCCCATAACTCGTGGGGGTAGCTATACTTGAGCTGTATCCG

**ZZ12 MH730693**

GGCATTCTAACTTAAACTACTTTCTGCATTCTTATGTTGTATAAGCTTCATAAAATAACCCTGACACCAACCTACTCATAATACTACTATGTAATTCGTGCATTACTGCTAGCCAGCATGTATAATATATAGTACTATATATGTTTAACTGTACATAGCGCATATCATTACATATCAACTTAACATTCTAAACAACATGCTTACAAGCAAGTACTTTGATACAAACCTCAACAGTAACACATAACACGATCTCTCCAAATTTAACTTGTTCTCCTCATGGATATCAACTAAACCAATCCATGCCAGTCGTCCATAGTACATTAAGTCGTTCATCGGACATAGTACATATTCATTAAATAATCCTCCTCACCACGGATGCCCCCCCTCACTTAGGAGTCCCTTGCTCACCATCCTCCGTGAAATCAATATCCCGCACAAGAGTGCTACTCTCCTCGCTCCGGGCCCATAACTCGTGGGGGTAGCTATACTTGAGCTGTATCCG

**ZZ13 MH730729**

GGCATTCTAACTTAAACTACTTTCTGTATTCTTATGTTGCATAACTTCATAAAATAACCCTAACATCAACCCACCCATAATATTACTATGTAATTCGTGCATTACTGCTAGCCAACATGTATAATATATAGTACTATATATGCTTAACTGTACATAACACATATCATTACATATCAACCCAACATTCCAGACAACATGCTTACAAGCAAGTACTCTAATACAAGTCCCAACAGTAATACATAGTATGGCCCTTCCAAATTCAATCTGTCCCCCCTCATGGATATCAACCAAACCAGTCCATGCCAGTCGTCCATAACACATTAAATTGTTCATCGGACATAGTACATATTCATTAAATAATCCTCCTCACCACGGATGCCCCCCCTCACTTAGGAGTCCCTTGTTCACCATCCTCCGTGAAATCAATATCCCGCACAAGAGTGCTACTCTCCTCGCTCCGGGCCCATAACTCGTGGGGGTAGCTATACTTGAGCTGTATCCG

**ZZ14 MH730731**

GGCATTCTAACTTAAACTACTTTCTGTATTCTTATGTTGCATAACTTCATAAAATAACCCTAACATCAACCCACCCATAATATTACTATGTAATTCGTGCATTACTGCTAGCCAACATGTATAATATATAGTACTATATATGTTTAACTGTACATAACACATATCGTTACATATCAACTCAAAATTCCAGACAACATGCTTACAAGCAAGTACTCTAATACAAGTTCCAACAGTAATACATAGTATGGCTCCTTCAAATTCAATCCGTCCCCCCCATGGATATCAACCGAACCAGTCCATGCCAGTCGTCCATAGTACATTAAATTGTTCATCGGACATAGTACATATTCATTAAATAATCCTCCTCACCACGGATGCCCCCCTTCACTTAGGAGTCCCTTGTTCACCATCCTCCGTGAAATCAATATCCCGCACAAGAGTGCTACTCTCCTCGCTCCGGGCCCATAACTCGTGGGGGTAGCTATACTTGAACTGTATCCG

**ZZ15 MH730738**

GGCATTCTAACTTAAACTACTTTCTGTATTCTTACGTTGCACAACTTCATAAAATAACCCTAACATTCAACCTACCCATAATATTACTATGTAATTCGTGCATTACTGCTAGCCAGCATGTATAATATATAGTACTATATATGCTTAACTGTACATAACACATATCATTACATATCAACTCAACATCCTAGACAACATGCTTACAAGCAAGTACTCTAATACAAGCCCCAACAGTAGCACATAATATGGCCTCTCCAAATTCAATTCACCTCCTCATGAATACCAACCGAACCAGTCCATGCCAGTCGTCCATAGTACATTAAATCGTTCATCGGACATAGCACATATTCATTAAATAATCCTCCTCACCACGGATGCCCCCCCTCACTTAGGAATCCCTTGTTCACCATCCTCCGTGAAATCAATATCCCGCACAAGAGTGCTACTCTCCTCGCTCCGGGCCCATAACTCGTGGGGGTAGCTATACTCGAGCTGTATCCG

**FZ1 MH730746**

GGCATTCTAACTTAAACTACTTTCTGTATTCTTATGTTGCATAACTTCATAAAATAACCCTAACATCAACCCACCCATAATATTACTATGTAATTCGTGCATTACTGCTAGCCAACATGTATAATATATAGTACTATATATGCTTAACTGTACATAACACATATTATTGCACATCAACCCAACATTCCAGACAACATGCTTACAAGCAAGTACTCTAATACAAGTCCCAACAGTAATACATAGTATGGCCCTTCCAAATTCAATCCGTCCCCCCATGAATATCAACCGAACCAGTTCATGCCAGTCGTCCATAGTACATTAAATTGTTCATCGGACATAGTACATATTCATTAAATAATCCTCCTCACCACGGATGCCCCCCCTCACTTAGGAGTCCCTTGCTCACCATCCTCCGTGAAATCAATATCCCGCACAAGAGTGCTACTCTCCTCGCTCCGGGCCCATAACTCGTGGGGGTAGCTATACTTGAGCTGTATCCG

**FZ2 MH730733**

GGCATTCTAACTTAAACTACTTTCTGCATTCTTATGTTGTATAAACTTCATAAAATAACCCTGATACAACCTACTCATAATACTACTATGTAATTCGTGCATTACTGCTAGCCAACATGTATAATATATAGTACTATATATGTTTAACTGTACATAACACATATCATTACATATCAACTTAACATTCTAGATAACATGCTTACAAGCAAGTACTTTGATATAGATCTCAACAGTAATACATAACATGGCTTCTCCAAATTCAATTTGTCCCCCTCATGAGTATCAACTAAACCAGTCCATGCCAGTCGTCCATAGTACATTAAATTGTTCATCGGACATAGCACATATTCATTAAATAATCCTCCTCACCACGGATGCCCCCCCTCACTTAGGAATCCCTTGCTCACCATCCTCCGTGAAATCAATATCCCGCACAAGAGTGCTACTCTCCTCGCTCCGGGCCCATAACTCGTGGGGGTAGCTATACTTGAGCTGTATCCG

**FZ3 MH730747**

GGCATTCTAACTTAAACTACTTTCTGTATTCTTATGTTGCATAACTTCATAAAATAACCCTAACATCAACCCACCCATAATATTACTATGTAATTCGTGCATTACTGCTAGCCAACATGTATAATATATAGTACTATATATGCTTAACTGTACATAACACATATCATTACATATCAACCCAACATTCCAGACAACATGCTTACAAGCAAGTACTCTAATACAAGTCCCAACAGTAATACATAGTATGGCCCTTCCAAATTCAATTCATCCCCCCATGGATATCAACCGAACCAGTCCATGCCAGTCGTCCATAGTACATTAAATTGTTCATCGGACATAGTACATATTCATTAAATAATCCTCTTCACCACGGGTGCCCCCCCTCACTTAGGAGTCCCTTGCTCACCATCCTCCGTGAAATCAATATCCCGCACAAGAGTGCTACTCTCCTCGCTCCGGGCCCATAACTCGTGGGGGTAGCTATACTTGAGCTGTATCCG

**LZ1 MH730701**

GGCATTCTAACTTAAACTACTTTCTGCATTCTTATGTTGTATAAGCTTCATAAAATAACCCTGACACCAACCTACTCATAATACTACTATGTAATTCGTGCATTACTGCTAGCCAACATGTATAATATATAGTACTATATATGTTTAACTGTACATAACACATATCATTACATATCAACTTAACATTCTAAACAACATGCTTACAAGCAAGTACTTTGATACAGACTCCAACAGTAACACATAACACGATCTCTCCAAATTTAACTTGTTCTCCTCATGGATATCAACTAAACCAGTCCATGCCAGTCGTCCATAGTACATTAAGTCGTTCATCGGACATAGCACATATTCATTAAATAATCCTCCTCACCACGGATGCCCCCCCTCACTTAGGAATCCCTTGCTCACCATCCTCCGTGAAATCAATATCCCGCACAAGAGTGCTACTCTCCTCGCTCCGGGCCCATAACTCGTGGGGGTAGCTATACTTGAGCTGTATCCG

**LZ2 MH730685**

GGTATTCTAACTTAAACTACTTTCTGCATTCTTATGTTGTATAAGCCTCATAAAATAACCCTAACACCAACCTACTCATAATATTACTATGTAATTCGTGCATTACTGCTAGCCAACATGTATAATATATAGTACTATATATGCTTAACTGTACATAACACATATCATTACATATCAACCCAACATTCTAGACAACATGCTTACAAGCAAGTACTCTAATATAAACTCCAACAGTAATACATAACATGACCTTTTCAAACTTGATCCGTCCTCCTCATAGATACCAACCGAACCAGCCCATGCCAGTCGTCCATAGTACATTAAATCGTTCATCGGACATAGTACATATTCATTAAATAATCCTCCTCACCACGGATGCCCCCCCTCACTTAGGAGTCCCTTGTTCACCATCCTCCGTGAAATCAATATCCCGCACAAGAGTGCTACTCTCCTCGCTCCGGGCCCATAACTCGTGGGGGTAGCTATACTTGAATTGTATCCG

**LZ3 MH730734**

GGCATTCTAACTTAAACTACTTTCTGTATTCTTATGTTGCACAACTTCATAAAATAACCCTAACATTCAACCTACCCATAATATTACTATGTAATTCGTGCATTACTGCTAGCCAACATGTATAATATATAGTACTATATATGCTTAACTGTACATAACACATATCATTACATATCAACCCAACATCCTAGACAACATGCTTACAGGCAAGTACTCTAATACAAGCTCCAACAGTAGTACATAATATGGCCTCTCCAAATTCAATTCACCCCCCCATGAATACCAACCGAACCAGTCCATGCCAATCGTCCATAGTACATTAGATCGTTCATCGGACATAGCACATATTCATTAAATAATCCTCCTCACCACGGATGCCCCCCCTCACTTAGGAATCCCTTGTTCACCATCCTCCGTGAAATCAATATCCCGCACAAGAGTGCTACTCTCCTCGCTCCGGGCCCATAACTCGTGGGGGTAGCTATATTTGAGCTGTATCCG

**LZ4^2^ MH730724**

GGCATTCTAACTTAAACTACTTTCTGCATTCTTATGTTGTATAAACTTCATAAAATAACCCTGACACCAACCTACTCATAATACTGCTATGTAATTCGTGCATTACTGCTAGTCAGCATGTATAATATATAGTACTATATATGTTTAACTGTACATAACACATATTATTACATATCAACTTAACATTCTAAACAACATGCTTACAAGCAAGTACCTCAATACAAACCTCAACAGTAACACATAACATGATTCCTTCAAATTCAACTTGTCCTCCTCATGAGTATCAACTAAACCAGTCCATGCCAGTCGTCCATAGTACATTAAATTGTTCATCGGACATAGCACATATTCATTAAATAATCCTCCTCACTACGGATGCCCCCCCTCACTTAGGAGTCCCTTGTTCACCATCCTCCGTGAAATCAATATCCCGCACAAGAGTGCTACTCTCCTCGCTCCGGGCCCATAACTCGTGGGGGTAGCTATACTTGAGCTGTATCCG

**NJ1^3^  MH730717**

GGCATTCTAACTTAAACTACTTTCTGCATTCTTATGTTGTATAAACTTCATAAAATAACCCTGACACCAACCTACTCATAATACTGCTATGTAATTCGTGCATTACTGCTAGTCAGCATGTATAATATATAGTACTATATATGTTTAACTGTACATAACACATATTATTACATATCAACTTAACATTCTAAACAACATGCTTACAAGCAAGTACCTCAATACAAATCTCAACAGTAACACATAACATGATTCTTTCAAATTCAACTTGTCCTCCTCATGAGTATCAACTAAACCAGTCCATGCCAGTCGTCCATAGTACATTAAATTGTTCATCGGACATAGCACATATTCATTAAATAATCCTCCTCACTACGGATGCCCCCCCTCACTTAGGGGTCCCTTGTTCACCATCCTCCGTGAAATCAATATCCCGCACAAGAGTGCTACTCTCCTCGCTCCGGGCCCATAACTCGTGGGGGTAGCTATACTTGAGCTGTATCCG

**NJ2 MH730707**

GGCATTCTAACTTAAACTACTTTCTGCATTCTTATGTTGTATAAACTTCATAAAATAACCCTGACACCAACCTACTCATAATACTGCTATGTAATTCGTGCATTACTGCTAGTCAGCATGTATAATATATAGTACTATATATGTTTAACTGTACATAACACATACTATTACATATCAACTTAACATTCCAAACAACATGCTTACAAGCAAGTACTTTAATACAAATCTCAACAGTAACACATAACATGGCTCCTTCAAACTCAACTTGTCCCCCTCATGAATATCAACTAGACCAGTCCATGCCAGTCGTCCATAGTACATTAAATTGTTCATCGGACATAGCACATATTCATTAAATAATCCTCCTCACCACGGATGCCCCCCCTCACTTAGGAATCCCTTGTTCACCATCCTCCGTGAAATCAATATCCCGCACAAGAGTGCTACTCTCCTCGCTCCGGGCCCATAACTCGTGGGGGTAGCTATACTTGAGCTGTATCCG

**NJ3 MH730720**

GGCATTCTAACTTAAACTACTTTCTGCATTCTTATGTTGTATAAACTTCATAAAATAACCCTGACACCAACCTACTCATAATACTGCTATGTAATTCGTGCATTACTGCTAGCCAGCATGTATAATATATAGTACTATATATGTTTAACTGTACATAACACATATTATTACATATCAACTTAACATTCTAAACAACATGCTTACAAGCAAGTACTTTAATACAAACCTCAACAGTAATACATAGCATGATTCCTTCAAATTCAACTTGTCCTCCTCATGAGTATCAACTAAACCAGTCCATGCCAGTCGTCCATAGTACATTAAATCGTTCATCGGACATAGCACATATTTATTAAATAATCCTCCTCACCACGGATGCCCCCCCTCACTTAGGAGTCCCTTGTTCACCATCCTCCGTGAAATCAATATCCCGCACAAGAGTGCTACTCTCCTCGCTCCGGGCCCATAACTCGTGGGGGTAGCTATACTTGAGCTGTATCCG

**NJ4 MH730723**

GGCATTCTAACTTAAACTACTTTCTGCATTCTTATGTTGTATAAACTTCATAAAATAACCCTGACACCAACCTACTCATAATACTGCTATGTAATTCGTGCATTACTGCTAGCCAGCATGTATAATATATAGTACTATATATGTTTAACTGTACATAACACATATTATTACATATCAACTTAACATTCTAAACAACATGCTTACAAGCAAGTACTTTAATACAAACCTCAACAGTAGTACATGGTATGATTCCTTCAAATTCAACTTGTCCTCCCCATGAGTATCAACTAAACCAGTCCATGCCAGTCGTCCATAGTACATTAAATTGTTCATCGGACATAGCACATATTCATTAAATAATCCTCCTCACCACGGATGCCCCCCCTCACTTAGGAGTCCCTTGTTCACCATCCTCCGTGAAATCAATATCCCGCACAAGAGTGCTACTCTCCTCGCTCCGGGCCCATAACTCGTGGGGGTAGCTATACTTGAGCTGTATCCG

**NJ5 MH730715**

GGCATTCTAACTTAAACTACTTTCTGCATTCTTATGTTGTATAAACTTCATAAAATAACCCTGACACCAACCTACTCATAATACTGCTATGTAATTCGTGCATTACTGCTAGCCAGCATGTATAATATATAGTACTATATATGTTTAACTGTACATAACACATATTATTACATATCAACTTAACATTCTAAACAACATGCTTACAAGCAAGTACTTTAATACAAACCTCAACAGTAATACATAGCATGATTCCTTCAAATTCAACTCGTCCTCCCCATGAGTATCAACTAAACCAGTCCATGCCAGTCGTCCATAGTACATTAAATTGTTCATCGGACATAGTACATATTCATTAAATAATCCTCCTCACCACGGATGCCCCCCCTCACTTAGGAGTCCCTTGTTCACCATCCTCCGTGAAATCAATATCCCGCACAAGAGTGCTACTCTCCTCGCTCCGGGCCCATAACTCGTGGGGGTAGCTATACTTGAGCTGTATCCG

**NJ6 MH730716**

GGCATTCTAACTTAAACTACTTTCTGCATTCTTATGTTGTATAAACTTCATAAAATAACCCTGACACCAACCTACTCATAATACTGCTATGTAATTCGTGCATTACTGCTAGTCAGCATGTATAATATATAGTACTATATATGTTTAACTGTACATAACACATATTATTACATATCAACTTAACATTCTAAACAACATGCTTACAAGCAAGTACTTTAATACAAATCTCAACAGTAACACATAACATGGCTTCTTCAAACTCAACTTGTCCCCCTCATGAATATTAACTAGACCAGTCCATGCCAGTCGTCCATAGTACATTAAATTGTTCATCGGACATAGCACATATTCATTAAATAATCCTCCTCACCACGGATGCCCCCCCTCACTTAGGAGTCCCTTGTTCACCATCCTCCGTGAAATCAATATCCCGCACAAGAGTGCTACTCTCCTCGCTCCGGGCCCATAACTCGTGGGGGTAGCTATACTTGAGCTGTATCCG

**NJ7 MH730710**

GGCATTCTAACTTAAACTACTTTCTGCATTCTTATGTTGTATAAACTTCATAAAATAACCCTGACACCAACCTACTCATAATACTGCTATGTAATTCGTGCATTACTGCTAGTCAGCATGTATAATATATAGTACTATATATGTTTAACTGTACATAACACATATTATTACATATCAACTTAACATTCTAAACAGCATGCTTACAAGCAAGTACCTCAATACAAACCTCAACAGTAACACATAACATGATTCCTTCAAATTCAACTTGTCCTCCTCATGAGTATCAACTAAACCAATCCATGCCAGTCGTCCATAGTACATTAAATTGTTCATCGGACATAGCACATATTCATTAAATAATCCTCCTCACTACGGATGCCCCCCCTCACTTAGGGGTCCCTTGTTCACCATCCTCCGTGAAATCAATATCCCGCACAAGAGTGCTACTCTCCTCGCTCCGGGCCCATAACTCGTGGGGGTAGCTATACTTGAGCTGTATCCG

**NJ8 MH730677**

GGCATTCTAACTTAAACTACTTTCTGCATTCTTATGTTGCATAAACCCCATAAAATAGCCCTGACACTAACTCACTCATAATACTACTATGTAATTCGTGCATTACTGCTAGCCAACATGTATAATATATAGTACTATATATGCTTAACTGTACATAATACATACCATCACATATCAACTTAACATCCTAAACAACATGCTTACAAGCAAGCACTTTAATACAGGCCTCAACAGCAGTACATAACATGGTCCTTTCAAACTTAACTTGTCTTCCTCATGAATATCAACCGAACCAGTCCATGCCAGTCGTCCATAGTACATTAAATCGTTCATTGGACATAGCACATATTCATTAAATAATCCTCCTCACCACGGATGCCCCTCCTCACTTAGGAGTCCCTTGTTCACCATCCTCCGTGAAATCAATATCCCGCACAAGAGTGCTACTCTCCTCGCTCCGGGCCCATAACTCGTGGGGGTAGCTATACCTGAACTGTATCCG

**NJ9 MH730698**

GGCATTCTAACTTAAACTACTTTCTGCATTCTTATGTTGTATAAACTTCATAAAATAACCCTGACACCAACCTACTCATAATACTGCTATGTAATTCGTGCATTACTGCTAGTCAGCATGTATAATATATAGTACTATATATGTTTAACTGTACATAACACATATTATTACATATCAACTTAACATTCTAAACAGCATGCTTACAAGCAAGTACCTCAATACAAACCTCAACAGTAACACATAACATGATTCCTTCAAATTCAACTTGTCCTCCTCATGAGTATCAACTAAACCAATCCATGCCAGTCGTTCATAGTACATTAAATTGTTCATCGGACATAGCACATATTCATTAAATAATCCTCCTCACTACGGATGCCCCCCCTCACTTAGGGGTCCCTTGTTCACCATCCTCCGTGAAATCAATATCCCGCACAAGAGTGCTACTCTCCTCGCTCCGGGCCCATAACTCGTGGGGGTAGCTATACTTGAGCTGTATCCG

**XA1 MH730739**

GGCATTCTAACTTAAACTACTTTCTGTATTCTTATGTTGCACAACTTCATAAAATAACCCTAACATTCAACCTACCCATAATATTACTATGTAATTCGTGCATTACTGCTAGCCAACATGTATAATATATAGTACTATATATGCTTAACTGTACATAACACATATCATTACATATCAACTCAACATCCTAGACAACATGCTTACAAGCAAGTACTCTAATACAAGTCCCAACAGTAGTACATAACATGGCTTCTCCAAATTCAATTCACCTCCTCATGAATACCAACCGAACCAGTCCATGCCAGTCGTCCATAGTACATTAAATCGTTCATCGGACATAGCACATATTCATTAAATAATCCTCCTCACCACGGATGCCCCCCCTCACTTAGGAATCCCTTGTTCACCATCCTCCGTGAAATCAATATCCCGCACAAGAGTGCTACTCTCCTCGCTCCGGGCCCATAACTCGTGGGGGTAGCTATACTCGAGCTGTATCCG

**XA2 MH730694**

GGCATTCTAACTTAAACTACTTTCTGCATTCTTATGTTGTATAAACTTCATAAAACAACCCTAACACCAACCTACTCATAATACTACTATGTAATTCGTGCATTACTGCTAGCCAACATGTATAATATATAGTACTATATATGCTTAACTGTACATAACACATACTATTACATATCAACTTAACATTCTAAACAACATGCTTACAAGCAAGTACTCTAATATAAACTTCAACAGTAACACATAATATGGCTTCTCCAAATTTGACCTATCTCCCTCATGAATATCAACCAAACCAGTTCATGCCAGTCGTCCATAGTACATTAAATCGTTCATCGGACATAGCACATATTCATTAAATAATCCTCCTCACCACGGATGCCCCCCCTCACTTAGGAATCCCTTATTCACCATCCTCCGTGAAATCAATATCCCGCACAAGAGTGCTACTCTCCTCGCTCCGGGCCCATAACTCGTGGGGGTAGCTATACTTGAACTGTATCCG

**XA3^1^  MH730741**

GGCATTCTAACTTAAACTACTTTCTGTATTCTTACGTTGCACAACTTCATAAAATAACCCTAACATTCAACCTACCCATAATATTACTATGTAATTCGTGCATTACTGCTAGCCAACATGTATAATATATAGTACTATATATGCTTAACTGTACATAACACATATCATTACATATCAACTCAACATCCTAGACAACATGCTTACAAGCAAGTACTCTAATACAAGCCCCAACAGTAGCACATAATATGGCCTCTCCAAATTCAATTCACCTCCTCATGAATACCAACCGAACCAGTCCATGCCAGTCGTCCATAGTACATTAAATCGTTCATCGGACATAGCACATATTCATTAAATAATCCTCCTCACCACGGATGCCCCCCCTCACTTAGGAATCCCTTGTTCACCATCCTCCGTGAAATCAATATCCCGCACAAGAGTGCTACTCTCCTCGCTCCGGGCCCATAACTCGTGGGGGTAGCTATACTCGAGCTGTATCCG

**XA4^6^ MH730745**

GGCATTCTAACTTAAACTACTTTCTGCATTCTTATGTTGTATAAACTTCATAAAATAACCCTGACACCAACCTACTCATAATACTGCTATGTAATTCGTGCATTACTGCTAGCCAACATGTATAATATATAGTACTATATATGCTTAACTGTACATAACACATATCATTACATATCAACTTAACATTCTAAACAACATGCTTACAAGCAAGTACTTTAATACGAACCTCAACAGTAGTACATAACATGATTTCTTCAAATTCAACTTGTCCCTCCCCATGAGTATCAACTAGACCAATCCATGCCAGTCGTCCATAGTACATTAAATCGTTCATCGGACATAGCACATATTCATTAAATAATCCTCCTCACTACGGATGCCCCCCCTCACTTAGGAGTCCCTTGTTCACCATCCTCCGTGAAATCAATATCCCGCACAAGAGTGCTACTCTCCTCGCTCCGGGCCCATAACTCGTGGGGGTAGCTATACTTGAGCTGTATCCG

**XA5 MH730709**

GGCATTCTAACTTAAACTACTTTCTGCATTCTTATGTTGTATAAACTTCATAAAATAACCCTGACACCAACCTACTCATAATACTGCTATGTAATTCGTGCATTACTGCTAGTCAGCATGTATAATATATAGTACTATATATGTTTAACTGTACATAACGCATACTATTACATATCAACTTAACATTCTAAGCAACATGCTTACAAGCAAGTACTTTAATACAAACCTCAACAGTAGTACATAGCATGATTCCTTCAAATTCAACTTGTCCTCCCCATGAGTATTAACTAAACCAGTCCATGCCAGTCGTCCATAGTACATTAAATTGTTCATCGGACATAGCACATATTCATTAAATAATCCTCCTCACCACGGATGCCCCCCCTCACTTAGGAGTCCCTTGTTCACCATCCTCCGTGAAATCAATATCCCGCACAAGAGTGCTACTCTCCTCGCTCCGGGCCCATAACTCGTGGGGGTAGCTATACTTGAGCTGTATCCG

**XA6 MH730690**

GGCATTCTAACTTAAACTACTTTCTGCATTCTTATGTTGCATAAACTCCATAAAATAGCCCTGACACTAACTCACTCATAATACTACTATGTAATTCGTGCATTACTGCTAGCCAACATGTATAATATATAGTACTATATATGTTTAATTGTACATAACACATACCATCACATATCAACTTAACATCCTAAACAACATGCTTACAAGCAAGTACTTTAATACAGGCCTCAACAGCAATACATAACATGGTCCTTTCAAATTTAACTTGTCTTCCTCATGAATATCAACCAAACCAGTCCATGCCAGTCGTCCATAGTACATTAAATCGTTCATCGGACATAGCACATATTCATTAAATAATCCTCCTCACCACGGATGCCCCCCCTCACTTAGGAGTCCCTTGTTCACCATCCTCCGTGAAATCAATATCCCGCACAAGAGTGCTACTCTCCTCGCTCCGGGCCCATAACTCGTGGGGGTAGCTATACCTGAACTGTATCCG

**XN1 MH730687**

GGTATTCTAACTTAAACTACTTTCTGCATTCTTATGTTGTATAAGCCTCATAAAATAACCCTAACACCAACCTACTCATAATATTACTATGTAATTCGTGCATTACTGCTAGCCAACATGTATAATATATAGTACTATATATGCTTAACTGTACATAACACATACCATTACATATCAACCCAACATTCTAGACAACATGCTTACAAGCAAGTACTCTAATATAAACTCCAACAGTAATACATAACATGACCTTTTCAAACTTAATCCGTCCTCCTCATAGATACCAACCGAACCAGTCCATGCCAGTCGTCCATAGTACATTAAATCGTTCATCGGACATAGTACATATTCATTAAATAATCCTCCTCACCACGGATGCCCCCCCTCACTTAGGAGTCCCTTGTTCACCATCCTCCGTGAAATCAATATCCCGCACAAGAGTGCTACTCTCCTCGCTCCGGGCCCATAACTCGTGGGGGTAGCTATACTTGAATTGTATCCG

**XN2 MH730680**

GGCATTCTAACTTAAACTACTTTCTGCATTCTTATGTTGCATAAACTCCATAAAATAGCCCTGACACTAACTCACTCATAATACTACTATGTAATTCGTGCATTACTGCTAGCCAACATGTATAATATATAGTACTATATATGCTTAACTGTACATAATACATACCATTACATATCAACTTAACATCCTAAACAACATGCTTACAAGCAAGTACTTTAATACGGGTCTCAACAGCAGTACATAACATGGTCTTTTCAAACTTAACTTGTCTTCCTCATGAATATCAACCGAACCAGTCCATGCCAGTCGTCCATAGTACATTAAATTGTTCATCGGACATAGCACATATTCATTAAATAATCCTCCTCACCACGGATGCCCCTCCTCACTTAGGAATCCCTTGTTCACCATCCTCCGTGAAATCAATATCCCGCACAAGAGTGCTACTCTCCTCGCTCCGGGCCCATAACTCGTGGGGGTAGCTATACTTGAACTGTATCCG

**XN3^7^  MH730726**

GGCATTCTAACTTAAACTACTTTCTGCATTCTTATGTTGTATAAACTTCATAAAATAACCCTGACACCAACCTACTCATAATACTGCTATGTAATTCGTGCATTACTGCTAGCCAGCATGTATAATATATAGTACTATATATGTTTAACTGTACATAACACATATTATTACATATCAACTTAACATTCTAAACAACATGCTTACAAGCAAGTACTTTAATACAAACCTCAACAGTAATACATAGCATGATTCCTTCAAATTCAACTTGTCCTCCTCATGAGTATCAACCAAACCAGTCCATGCCAGTCGTCCATAGTACATTAAATCGTTCATCGGACATAGCACATATTCATTAAATAATCCTCCTCACCACGGATGCCCCCCCTCACTTAGGAGTCCCTTGTTCACCATCCTCCGTGAAATCAATATCCCGCACAAGAGTGCTACTCTCCTCGCTCCGGGCCCATAACTCGTGGGGGTAGCTATACTTGAGCTGTATCCG

**XN4 MH730675**

GGCATTCTAACTTAAACTACTTTCTGCATTCTTATGTTGCATAAACTCCATAAAATAGCCCTGACACTAACTCACTCATAATACTACTATGTAATTCGTGCATTACTGCTAGCCAGCATGTATAATATATAGTACTATATATGCTTAATCGTACATAATACATACCATCACATATCAACTTAACATCCTAAACAACATGCTTACAAGCAAGCACTTTAATACGGGCCTCAACAGCAGTACATAACATGGTCTTTTCAAACTTAACTTGTCTTCCTCATGAATATCAACCGAACCAGTCCATGCCAGTCGTCCATAGTACATTAAATCGTTCATCGGACATAGCACATATTCATTAAATAATCCTCCGCACCACGGATGCTCCTCTTCACTTAGGGGTCCCTTGTTCACCATCCTCCGTGAAATCAATATCCCGCACAAGAGTGCTACTCTCCTCGCTCCGGGCCCATAACTCGTGGGGGTAGCTATACCTGAACTGTATCCG

**XN5 MH730732**

GGCATTCTAACTTAAACTACTTTCTGCATTCTTATGTTGTATAAACTTCATAAAATAACCCTGATACAACCTACTCATAATACTACTATGTAATTCGTGCATTACTGCTAGCCAACATGTATAATATATAGTACTATATATGTTTAACTGTACATAACACATATCATTACATATCAACTTAATATTCTAGACAACATGCTTACAAGCAAGTACTTTGATATAGATCTCAACAGTAATACATAACATGGCCTCTCCAAATTCAACTTGTCCTCCTCATGAGTATCAACTAAACCAATCCATGCCAGTCGTCCATAGTACATTAAATTGTTCATCGGACATAGCACATATTTATTAAATAATCCTCCTCACCACGGATGCCCCCCCTCACTTAGGAATCCCTTGCTCACCATCCTCCGTGAAATCAATATCCCGCACAAGAGTGCTACTCTCCTCGCTCCGGGCCCATAACTCGTGGGGGTAGCTATACTTGAGCTGTATCCG

**XN6 MH730695**

GGCATTCTAACTTAAACTACTTTCTGCATTCTTATGTTGTATAAACTTCATAAAATAACCCTAACACCAACCTACTCATAATACTACTATGTAATTCGTGCATTACTGCTAGCCAACATGTATAATATATAGTACTATATATGCTTAACTGTACATAACACATACTATTACATATCAACTTAACATTCTAGACAACATGCTTACAAGCAAGTACTCTAATATAAACTTCAACAGTAACACATAATATGGCTTCTCCAAATTTGACCTATCTCCCTCATGAATATCAACCAAACCAGTTCATGCCAGTCGTCCATAGTACATTAAATCGTTCATCGGACATAGCACATATTCATTAAATAATCCTCCTCACCACGGATGCCCCCCCTCACTTAGGAATCCCTTATTCACCATCCTCCGTGAAATCAATATCCCGCACAAGAGTGCTACTCTCCTCGCTCCGGGCCCATAACTCGTGGGGGTAGCTATACTTGAGCTGTATCCG

**XN7 MH730704**

GGCATTCTAACTTAAACTACTTTCTGCATTCTTATGTTGTATAAGCTTCATAAAATAACCCTGACACCAACCTACTCATAATACTACTATGTAATTCGTGCATTACTGCTAGCCAACATGTATAATATATAGTACTATATATGTTTAACTGTACATAACACATATCATTACATATCAACTTAACATTCTAAACAACATGCTTACAAGCAAGTACTTTGATACAGACCCCAACAGTAACACATAACACGATCTCTCCAAATTCAACTTGTCCTCCTCATGGATATCAACTAAACCAGTCCATGCCAGTCGTCCATAGTACATTAAGTCGTTCATCGGACATAGCACATATTCATTAAATAATCCTCCTCACCACGGATGCCCCCCCTCACTTAGGAATCCCTTGCTCACCATCCTCCGTGAAATCAATATCCCGCACAAGAGTGCTACTCTCCTCGCTCCGGGCCCATAACTCGTGGGGGTAGCTATACTTGAGCTGTATCCG

**HEB1^5^ MH730743**

GGCATTCTAACTTAAACTACTTTCTGCATTCTTATGTTGTATAAACTTCATAAAATAACCCTGACACCAACCTACTCATAATACTGCTATGTAATTCGTGCATTACTGCTAGCCAACATGTATAATATATAGTACTATATATGCTTAACTGTACATAACACATATCATTACATATCAACTTAACATTCTAAACAACATGCTTACAAGCAAGTACTTTAATACGAACCTCAACAGTAGTACATAACATGATTTCTTCAAATTCAACTTGTCCCTCCCCATGAGTATCAACTAGACCAACTCATGCCAGTCGTCCATAGTACATTAAATCGTTCATCGGACATAGCACATATTCATTAAATAATCCTCCTCACTACGGATGCCCCCCCTCACTTAGGAGTCCCTTGTTCACCATCCTCCGTGAAATCAATATCCCGCACAAGAGTGCTACTCTCCTCGCTCCGGGCCCATAACTCGTGGGGGTAGCTATACTTGAGCTGTATCCG

**HEB2 MH730748**

GGCATTCTAACTTAAACTACTTTCTGTATTCTTATGTTGCATAACTTCATAAAATAACCCTAATATCAACCCACCCATAATATTACTATGTAATTCGTGCATTACTGCTAGCCAACATGTATAATATATAGTACTATATATGCTTAACTGTACATAACACATACCATTACATATCAACCCAACATCCCAGACAACATGCTTACAAGCAAGTACTCTAATACAAGTCCCAACAGTAATACATAGTATGGTCCTTCCAAATTCAATCCATCCCCCCATGAATATCAACCGAACCAGTCCATGCCAGTCGTCCATAGTACATTAAATTGTTCATCGGACATAGTACATATTCGTTAAATAATCCTCTCCACCACGGATGCCCCCCCTCACTTAGGAGTCCCTTGTTCACCATCCTCCGTGAAATCAATATCCCGCACAAGAGTGCTACTCTCCTCGCTCCGGGCCCATAACTCGTGGGGGTAGCTATACTTGAGCTGTATCCG

**HEB3 MH730689**

GGCATTCTAACTTAAACTACTTTCTGCATTCTTATGTTGCATAAACTCCATAAAATAGCCCTGACACTAACTTACTCATAATACTACTATGTAATTCGTGCATTACTGCTAGCCAACATGTATAATATATAGTACTATATATGCTTAACTGTACATAATACATATCATCACATATCAACTTAACATCCTAAACAACATGCTTACAAGCAAGTACTTTAATACAGGCCTCAACAGCAATACATAACATGGTCCTTTCAAACTTAACTTGTCTCCCTCATGAATATCAACCGAACCAGTCCATGCCAGTCGTCCATAGTACATTAAATCGTTCATCGGACATAGCACATATTCATTAAATAATCCTCCTCACCACGGATGCCCCCCCTCACTTAGGGGTCCCTTGTTCACCATCCTCCGTGAAATCAATATCCCGCACAAGAGTGCTACTCTCCTCGCTCCGGGCCCATAACTCGTGGGGGTAGCTATACCTGAACTGTATCCG

**HEB4 MH730719**

GGCATTCTAACTTAAACTACTTTCTGCATTCTTATGTTGTATAAACTTCATAAAATAACCCTGACACCAACCTACTCATAATACTGCTATGTAATTCGTGCATTACTGCTAGTCAGCATGTATAATATATAGTACTATATATGTTTAACTGTACATAACACATATTATTACATATCAACTTAACATTCTAAACAACATGCTTACAAGCAAGTACTTTAATACAAATCTCAACAGTAACACATAACATGGCTCCTTCAAACTCAACTTGTCCCCCTCATGAATATCAACTAGACCAGTCCATGCCAGTCGTCCATAGTACATTAAATTGTTCATCGGACATAGCACATATTCATTAAATAATCCTCCTCACCACGGATGCCCCCCCTCACTTAGGAGTCCCTTGTTCACCATCCTCCGTGAAATCAATATCCCGCACAAGAGTGCTACTCTCCTCGCTCCGGGCCCATAACTCGTGGGGGTAGCTATACTTGAGCTGTATCCG

**HEB5 MH730706**

GGCATTCTAACTTAAACTACTTTCTGCATTCTTATGTTGTATAAACTTCATAAAATAACCCTGACACCAACCTACTCATAATACTGCTATGTAATTCGTGCATTACTGCTAGCCAGCATGTATAATATATAGTACTATATATGTTTAACTGTACATAACACATATTATTACATATCAACTTAACATTCTAAACAACATGCTTACAAGCAAGTACTTTAATACAAATCTCAACAGTAACACATAACATGGCTCCTTCAAACTCAACTTGTCCCCCTCATGAATATCAACTAGACCAGTCCATGCCAGTCGTCCATAGTACATTAAATTGTTCATCGGACATAGCACATATTCATTAAATAATCCTCCTCACTACGGATGCCCCCCCTCACTTAGGAGTCCCTTGTTCACCATCCTCCGTGAAATCAATATCCCGCACAAGAGTGCTACTCTCCTCGCTCCGGGCCCATAACTCGTGGGGGTAGCTATGCTTGAGCTGTATCCG

**HEB6^6^ MH730744**

GGCATTCTAACTTAAACTACTTTCTGCATTCTTATGTTGTATAAACTTCATAAAATAACCCTGACACCAACCTACTCATAATACTGCTATGTAATTCGTGCATTACTGCTAGCCAACATGTATAATATATAGTACTATATATGCTTAACTGTACATAACACATATCATTACATATCAACTTAACATTCTAAACAACATGCTTACAAGCAAGTACTTTAATACGAACCTCAACAGTAGTACATAACATGATTTCTTCAAATTCAACTTGTCCCTCCCCATGAGTATCAACTAGACCAATCCATGCCAGTCGTCCATAGTACATTAAATCGTTCATCGGACATAGCACATATTCATTAAATAATCCTCCTCACTACGGATGCCCCCCCTCACTTAGGAGTCCCTTGTTCACCATCCTCCGTGAAATCAATATCCCGCACAAGAGTGCTACTCTCCTCGCTCCGGGCCCATAACTCGTGGGGGTAGCTATACTTGAGCTGTATCCG

**HEB7 MH730692**

GGCATTCTAACTTAAACTACTTTCTGCATTCTTATGTTGTATAAACTTCATAAAACAACCCTAACACCAACCTACTCATAATACTACTATGTAATTCGTGCATTACTGCTAGCCAACATGTATAATATATAGTACTATATATGCTTAACTGTACATAACACATACTATTACATATCAACTTAACATTCTAGACAACATGCTTACAAGCAAGTACTCTAATATAAACTTCAGCAGTAACACATAATATGGCTTCTCCAAATTTGACCTGTCCTCCTCATGAATATCGACCAAACCAGTTCATGCCAGTCGTCCATAGTACATTAAATCGTTCATCGGACATAGCACATATTCATTAAATAATCCTCCTCACCACGGATGCCCCCCCTCACTTAGGAATCCCTTATTCACCATCCTCCGTGAAATCAATATCCCGCACAAGAGTGCTACTCTCCTCGCTCCGGGCCCATAACTCGTGGGGGTAGCTATACTTGAGCTGTATCCG

**HEB8 MH730735**

GGCATTCTAACTTAAACTACTTTCTGTATTCTTATGTTGCACAACTTCATAAAATAACCCTAACATTCAACCTACCCATAATATTACTATGTAATTCGTGCATTACTGCTAGCCAACATGTATAATATATAGTACTATATATGCTTAACTGTACATAACACATACCATTACATATCAACTCAACATCCTAGACAACATGCTTACAAGCAAGTACTCTAATACAAGTTCCAACAGTAGTACATAATATGGCCTCTCCAAGTTCAATTCACTTCCTCATGGATACCAACCGAACCAGTCCATGCCAATCGTCCATAGTACATTAAATCGTTCATCGGACATAGCACATATTCATTAAATAATCCTCCTCACCACGGATGCCCCCCCTCACTTAGGAGTCCCTTGTTCACCATCCTCCGTGAAATCAATATCCCGCACAAGAGTGCTACTCTCCTCGCTCCGGGCCCATAACTCGTGGGGGTAGCTATACTCGAGCTGTATCCG

**HEB9 MH730730**

GGCATTCTAACTTAAACTACTTTCTGCATTCTTATGTTGTATAAACTTCATAAAATAACCCTGACACAACCTACTCATAATACTGCTATGTAATTCGTGCATTACTGCTAGCCAGCATGTATAATATATAGTACTATATATGTTTAACTGTACATAACACATATTATTACATATCAACTTAACATTCTAAACAACATGCTTACAAGCAAGTACTTTAATACAAATCTCAACAGTAATACATAGCATGATTCTTTCAAATTCAACCTGTCCTCCCCATGAGTATCAACTAAACCAGTCCATGCCAGTCGTCCATAGTACATTAAATTGTTCATCGGACATAGTACATATTCATTAAATAATCCTCCTCACCACGGATGCCCCCCCTCACTTAGGAGTCCCTTGTTCACCATCCTCCGTGAAATCAATATCCCGCACAAGAGTGCTACTCTCCTCGCTCCGGGCCCATAACTCGTGGGGGTAGCTATACTTGAGCTGTATCCG

**HEB10 MH730712**

GGCATTCTAACTTAAACTACTTTCTGCATTCTTATGTTGTATAAACTTCATAAAATAACCCTGACACCAACCTACTCATAATACTGCTATGTAATTCGTGCATTACTGCTAGCCAGCATGTATAATATATAGTACTATATATGTTTAACTGTACATAACACATATTATTACATATCAACTTAACATTCTAATCAACATGCTTACAGGCAAGTACCTCAATACAGATCTCAACAGTAACACATAACATGATTCCTTCAAATTCAACTTGTCCTCCTCATGAGTATCAACTAAACCAGTCCATGCCAGTCGTCCATAGTACATTAAATTGTTCATCGGACATAGCACATATTCATTAAATAATCCTCCTCACTACGGATGCCCCCCCTCACTTAGGAGTCCCTTGTTCACCATCCTCCGTGAAATCAATATCCCGCACAAGAGTGCTACTCTCCTCGCTCCGGGCCCATAACTCGTGGGGGTAGCTATACTTGAGCTGTATCCG

**LS1^2^ MH730686**

GGTATTCTAACTTAAACTACTTTCTGCATTCTTATGTTGTATAAGCCTCATAAAATAACCCTAACACCAACCTACTCATAATATTACTATGTAATTCGTGCATTACTGCTAGCCAACATGTATAATATATAGTACTATATATGCTTAACTGTACATAACACATATCATTACATATCAACCCAACATTCTAGACAACATGCTTACAAGCAAGTACTCTAATATAAACTCCAACAGTAATACATAACATGACCTTTTCAAACTTGATCCGTCCTCCTCATAGATACCAACCGAACCAGCCCATGCCAGTCGTCCATAGTACATTAAATCGTTCATCGGACATAGTACATATTCATTAAATAATCCTCCTCACCACGGATGCCCCCCCTCACTTAGGAGTCCCTTGTTCACCATCCTCCGTGAAATCAATATCCCGCACAAGAGTGCTACTCTCCTCGCTCCGGGCCCATAACTCGTGGGGGTAGCTATACTTGAATTGTATCCG

**LS2 MH730688**

GGTATTCTAACTTAAACTACTTTCTGCATTCTTATGTTGTATAAGCCTCATAAAATAACCCTAACACCAACCTACTCATAATATTACTATGTAATTCGTGCATTACTGCTAGCCAACATGTATAATATATAGTACTATATATGTTTAACTGTACATAACACATATCATTACATATCAACCCAACATTCTAGACAACATGCTTACAAGCAAGTACTCTAATATAAACTCCAACAGTAATACATAACATGACCTTCTCAAACTTGATCCGTCCTCCTCATAGATACCAACCGAACCAGCCCATGCCAGTCGTCCATAGTACATTAAATCGTTCATCGGACATAGTACATATTCATTAAATAATCCTCCTCACCACGGATGCCCCCCCTCACTTAGGAGTCCCTTGTTCACCATCCTCCGTGAAATCAATATCCCGCACAAGAGTGCTACTCTCCTCGCTCCGGGCCCATAACTCGTGGGGGTAGCTATACTTGAATTGTATCCG

**NN1 MH730682**

GGCATTCTAACTTAAACTACTTTCTGCATTCTTATGTTGCATAAACTCCATAAAATAGCCCTGACACTAACTCACTCATAATACTACTATGTAATTCGTGCATTACTGCTAGCCAACATGTATAATATATAGTACTATATATGCTTAATTGTACATAATACATACCATCACATATCAACTTAACATCCTAAACAACATGCTTACAAGCAAGTACTTTAATACAGGCCTCAACAGCAATACATAACATGGTCCTTTCAAACTTAACTTGTCTTCCTCATGAATATCAACCAAACCAGTCCATGCCAGTCGTCCATAGTACATTAAATCGTTCATCGGACATAGCACATATTCATTAAATAATCCTCCTCACCACGGATGCCCCTCCTCACTTAGGAGTCCCTTGTTCACCATCCTCCGTGAAATCAATATCCCGCACAAGAGTGCTACTCTCCTCGCTCCGGGCCCATAACTCGTGGGGGTAGCTATACCTGAACTGTATCCG

**NN2 MH730681**

GGCATTCTAACTTAAACTACTTTCTGCATTCTTATGTTGCATAAACTCCATAAAATAGCCCTGACACTAACTTACTCATAATACTACTATGTAATTCGTGCATTACTGCTAGCCAGCATGTATAATATATAGTACTATATATGCTTAACTGTACATAATACATATCATCACATATCAACTTAACATCCCGAACAACATGCTTACAAGCAAGTACTCTAATACAGGCCTCAACAGCAGTACATAACATGGTCCTTTCAAACTTAACTTGTCTCCCTCATGAATATCAACCGAACCAGTCCATGCCAGTCGTCCATAGTACATTAAATCGTTCATCGGACATAGCACATATTCATTAAATAATCCTCCTCACCACGGATGCCCCCCCTCACTTAGGGGTCCCTTGTTCACCATCCTCCGTGAAATCAATATCCCGCACAAGAGTGCTACTCTCCTCGCTCCGGGCCCATAACTCGTGGGGGTAGCTATACCTGAACTGTATCCG

**NN3 MH730683**

GGCATTCTAACTTAAACTACTTTCTGCATTCTTATGTTGCATAAACTCCATAAAATAGCCCTGACACTAACTCACTCATAATACTACTATGTAATTCGTGCATTACTGCTAGCCAACATGTATAATATATAGTACTATATATGCTTAATTGTACATAATACATACCATCACATATCAACTTAACATCCTAAACAACATGCTTACAAGCAAGTACTTTAATACAGGCCTCAACAGTAATACATAACATGGTCCTTTCAAACTTAACTTGTCTTCCTCATGAATATCAACCAAACCAGTCCATGCCAGTCGTCCATAGTACATTAAATCGTTCATCGGACATAGCACATATTCATTAAATAATCCTCCTCACCACGGATGCCCCTCCTCACTTAGGAGTCCCTTGTTCACCATCCTCCGTGAAATCAATATCCCGCACAAGAGTGCTACTCTCCTCGCTCCGGGCCCATAACTCGTGGGGGTAGCTATACCTGAACTGTATCCG

**NN4^4^  MH730699**

GGCATTCTAACTTAAACTACTTTCTGCATTCTTATGTTGTATAAACTTCATAAAATAACCCTGACACCAACCTACTCATAATACTACTATGTAATTCGTGCATTACTGCTAGCCAACATGTATAATATATAGTACTATATATGCTTAACTGTACATAACACATACCATTACATATCAACCTAACATTCTAAACAACATGCTTACAAGCAAGTACTCTGATACAAACCTCAACAGTAATACATAACATGGTTCTTCCAAATTCAACTTGTCCTCCTCATGAATATCAACTAAACCAGTCCATGCCAGTCGTCCATAGTACATTAAATCGTTCATCGGACATAGTACATATTCATTAAATAATCCTCCTCACCACGGATGCCCCCCCTCACTTAGGGGTCCCTTGTTCACCATCCTCCGTGAAATCAATATCCCGCACAAGAGTGCTACTCTCCTCGCTCCGGGCCCATAACTCGTGGGGGTAGCTATACTTGAGCTGTATCCG

**GD1^7^ MH730669**

GGCATTCTAACTTAAACTACTTTCTGCATTCTTATGTTGTATAAACTTCATAAAATAACCCTGACACCAACCTACTCATAATACTGCTATGTAATTCGTGCATTACTGCTAGCCAGCATGTATAATATATAGTACTATATATGTTTAACTGTACATAACACATATTATTACATATCAACTTAACATTCTAAACAACATGCTTACAAGCAAGTACTTTAATACAAACCTCAACAGTAATACATAGCATGATTCCTTCAAATTCAACTTGTCCTCCTCATGAGTATCAACCAAACCAGTCCATGCCAGTCGTCCATAGTACATTAAATCGTTCATCGGACATAGCACATATTCATTAAATAATCCTCCTCACCACGGATGCCCCCCCTCACTTAGGAGTCCCTTGTTCACCATCCTCCGTGAAATCAATATCCCGCACAAGAGTGCTACTCTCCTCGCTCCGGGCCCATAACTCGTGGGGGTAGCTATACTTGAGCTGTATCCG

**GD2^4^  MH730670**

GGCATTCTAACTTAAACTACTTTCTGCATTCTTATGTTGTATAAACTTCATAAAATAACCCTGACACCAACCTACTCATAATACTACTATGTAATTCGTGCATTACTGCTAGCCAACATGTATAATATATAGTACTATATATGCTTAACTGTACATAACACATACCATTACATATCAACCTAACATTCTAAACAACATGCTTACAAGCAAGTACTCTGATACAAACCTCAACAGTAATACATAACATGGTTCTTCCAAATTCAACTTGTCCTCCTCATGAATATCAACTAAACCAGTCCATGCCAGTCGTCCATAGTACATTAAATCGTTCATCGGACATAGTACATATTCATTAAATAATCCTCCTCACCACGGATGCCCCCCCTCACTTAGGGGTCCCTTGTTCACCATCCTCCGTGAAATCAATATCCCGCACAAGAGTGCTACTCTCCTCGCTCCGGGCCCATAACTCGTGGGGGTAGCTATACTTGAGCTGTATCCG

**GD3 MH730671**

GGCATTCTAACTTAAACTACTTTCTGCATTCTTATGTTGCATAAACTCCATAAAATAGCCCTGACACTAACTTACTCATAATACTACTATGTAATTCGTGCATTACTGCTAGCCAACATGTATAATATATAGTACTATATATGCTTAACTGTACATAATACATACCATCACATATCAACTTAACATCCCAAACAACATGCTTACAAGCAAGTACTTTAATACAGGCCTCAACAGCAGTACATAACATGGTCCCTTCAAACTTAACTTATCTCCCTCATGAATATCAACCGAACCAGTCCATGCCAGTCGTCCATAGTACATTAAATCGTTCATCGGACATAGCACATATTCATTAAATAATCCTCCTCACCACGGATGCCCCCCCTCACTTAGGGGTCCCTTGTTCACCATCCTCCGTGAAATCAATATCCCGCACAAGAGTGCTACTCTCCTCGCTCCGGGCCCATAACTCGTGGGGGTAGCTATACCTGAACTGTATCCG

**GD4 MH730672**

GGCATTCTAACTTAAACTACTTTCTGCATTCTTATGTTGTATAAACTTCATAAAATAACCCTGACACCAACCTACTCATAATACTGCTATGTAATTCGTGCATTACTGCTAGTCAGCATGTATAATATATAGTACTATATATGTTTAACTGTACATAACACATATTATTACATATCAACTTAACATTCTAAACAACATGCTTACAAGCAAGTACTTTAATACAAATCTCAACAGTAACACATAACATGGCTCCTTCAAACCCAACTTGTCCCCCTCATGAGTATCAACTAAACCAGTCCATGCCAGTCGTCCATAGTACATTAAATTGTTCATCGGACATAGCACATATTCATTAAATAATCCTCCTCACCACGGATGCCCCCCCTCACTTAGGAGTCCCTTGTTCACCATCCTCCGTGAAATCAATATCCCGCACAAGAGTGCTACTCTCCTCGCTCCGGGCCCATAACTCGTGGGGGTAGCTATACTTGAGCTGTATCCG

**GD5 MH730673**

GGCATTCTAACTTAAACTACTTTCTGCATTCTTATGTTGTATAAACTTCATAAAATAACCCTGACACCAACCTACTCATAATACTACTATGTAATTCGTGCATTACTGCTAGCCAACATGTATAATATATAGTACTATATATGCTTAACTGTACATAACACATACCATTACATATCAACCTAACATTCTAAACAACATGCTTACAAGCAAGTACTCTGATACAAACCTCAACAGTAATACATAACATGGTTCTTCCAAATTCAACTTGTCCTCCTCATGAATATCAACTAAACCAGTCCATGCCAGTCGTCCATAGTACATTTAATCGTTCATCGGACATAGTACATATTCATTAAATAATCCTCCTCACCACGGATGCCCCCCCTCACTTAGGGGTCCCTTGTTCACCATCCTCCGTGAAATCAATATCCCGCACAAGAGTGCTACTCTCCTCGCTCCGGGCCCATAACTCGTGGGGGTAGCTATACTTGAGCTGTATCCG

**WLMQ1 MH730713**

GGCATTCTAACTTAAACTACTTTCTGCATTCTTATGTTGTATAAACTTCATAAAATAACCCTGACACCAACCTACTCATAATACTGCTATGTAATTCGTGCATTACTGCTAGTCAGCATGTATAATATATAGTACTATATATGTTTAACTGTACATAACACATATTATTACATATCAACTTAACATTCTAAACAACATGCTTACAAGCAAGTACCTCAATACAAACCTCAACAGTAACACATAGCATGATTCCTTCAAATTCAACTTGTCCTCCTCATGAATATCAACTAAACCAGTCCATGCCAGTCGTCCATAGTACATTAAATTGTTCATCGGACATAGCACATATTCATTAAATAATCCTTCTCACTACGGATGCCCCCCCTCACTTAGGGGTCCCTTGTTCACCATCCTCCGTGAAATCAATATCCCGCACAAGAGTGCTACTCTCCTCGCTCCGGGCCCATAACTCGTGGGGGTAGCTATACTTGAGCTGTATCCG

**WLMQ2 MH730705**

GGCATTCTAACTTAAACTACTTTCTGCATTCTTATGTTGTATAAGCTTCATAAAATAACCCTGACACCAACCTACTCATAATACTACTATGTAATTCGTGCATTACTGCTAGCCAGCATGTATAATATATAGTACTATATATGTTTAACTGTACATAACACATATCATTACATATCAACTTAACATTCTAAACAACATGCTTACAAGCAAGTACTTTGATACAAGCCCCAACAGTAACACATAACACGATCTCTCCAAATTTAACTTGTTCTCCTCATGGATATCAACCAAACCAGTCCATGCCAGTCGTCCATAGTACATTAAATCGTTCATCGGACATAGCACATATTCATTAAATAATCCTCCTCACCACGGATGCCCCCCCTCACTTAGGAATCCCTTGCTCACCATCCTCCGTGAAATCAATATCCCGCACAAGAGTGCTACTCTCCTCGCTCCGGGCCCATAACTCGTGGGGGTAGCTATACTTGAGCTGTATCCG

**WLMQ3 MH730721**

GGCATTCTAACTTAAACTACTTTCTGCATTCTTATGTTGTATAAACTTCATAAAATAACCCTGACACCAACCTACTCATAATACTGCTATGTAATTCGTGCATTACTGCTAGTCAGCATGTATAATATATAGTACTATATATGTTTAACTGTACATAACACATATTATTACATATCAACTTAACATTCTAAACAACATGCTTACAAGCAAGTACCTCAATACAAATCTCAACAGTAACACATAACATGATTCCTTCAAATTCAACTTGTCCTCCTCATGAGTATCAACTAAACCAGTCCATGCCAGTCGTCCATAGTACATTAAATTGTTCATCGGACATAGCACATATTCATTAAATAATCCTCCTCACTACGGATGCCCCCCCTCACTTAGGGGTCCCTTGTTCACCATCCTCCGTGAAATCAATATCCCGCACAAGAGTGCTACTCTCCTCGCTCCGGGCCCATAACTCGTGGGGGTAGCTATACTTGAGCTGTATCCG

**WLMQ4 MH730708**

GGCATTCTAACTTAAACTACTTTCTGCATTCTTATGTTGTATAAGCTTCATAAAATAACCCTGACACCAACCTACTCATAATACTACTATGTAATTCGTGCATTACTGCTAGCCAACATGTATAATATATAGTACTATATATGTTTAACTGTACATAACACATATCATTACATATCAACTTAACATTCTAAACAACATGCTTACAAGCAAGTACTTTGATACAGACCCCAACAGTAACACATAACATGATCTCTCCAAATTTAACTTGTCCTCCTCATGGATATCAACTAAACCAGTCCATGCCAGTCGTCCATAGTACATTAAGTCGTTCATCGGACATAGCACATATTCATTAAATAATCCTCCTCACCACGGATGCCCCCCCTCACTTAGGAATCCCTTGTTCACCATCCTCCGTGAAATCAATATCCCGCACAAGAGTGCTACTCTCCTCGCTCCGGGCCCATAACTCGTGGGGGTAGCTATACTTGAGCTGTATCCG

**WLMQ5 MH730697**

GGCATTCTAACTTAAACTACTTTCTGCATTCTTATGTTGTATAAGCTTCATAAAATAACCCTGACACCAACCTACTCATAATACTACTATGTAATTCGTGCATTACTGCTAGCCAGCATGTATAATATATAGTACTATATATGTTTAACTGTACATAACACATATCATTACATATCAACTTAACATCCTAAACAACATGCTTACAAGCAAGTACTTTGATACAGACCTCAACAGTAACACATAACACGATCCCTCCAAATTTAACTTGTTCTCCTCATGGATATCAACTAAACCAGTCCATGCCAGTCGTCCATAGTACATTAAGTCGTTCATCGGACATAGCACATATTCATTAAATAATCCTCCTCACCACGGATGCCCCCCCTCACTTAGGAATCCCTTGCTCACCATCCTCCGTGAAATCAATATCCCGCACAAGAGTGCTACTCTCCTCGCTCCGGGCCCATAACTCGTGGGGGTAGCTATACTTGAGCTGTATCCG

**WLMQ6 MH730722**

GGCATTCTAACTTAAACTACTTTCTGCATTCTTATGTTGTATAAACTTCATAAAATAACCCTGACACCAACCTACTCATAATACTGCTATGTAATTCGTGCATTACTGCTAGTCAGCATGTATAATATATAGTACTATATATGTTTAACTGTACATAACACATATTATTACATATCAACTTAACATTCTAAACAACATGCTTACAAGCAAGTACCTCAATACAAATCTCAACAGTAACACATAACATGATTCCTTCAAATTCAACTTGTCCTCCTCATGAGTATCAACTAAACCAGTCCATGCCAGTCGTCCATAGTACATTAAATCGTTCATCGGACATAGCACATATTCATTAAATAATCCTCCTCACTACGGATGCCCCCCCTCACTTAGGGGTCCCTTGTTCACCATCCTCCGTGAAATCAATATCCCGCACAAGAGTGCTACTCTCCTCGCTCCGGGCCCATAACTCGTGGGGGTAGCTATACTTGAGCTGTATCCG

**WLMQ7^3^ MH730718**

GGCATTCTAACTTAAACTACTTTCTGCATTCTTATGTTGTATAAACTTCATAAAATAACCCTGACACCAACCTACTCATAATACTGCTATGTAATTCGTGCATTACTGCTAGTCAGCATGTATAATATATAGTACTATATATGTTTAACTGTACATAACACATATTATTACATATCAACTTAACATTCTAAACAACATGCTTACAAGCAAGTACCTCAATACAAATCTCAACAGTAACACATAACATGATTCTTTCAAATTCAACTTGTCCTCCTCATGAGTATCAACTAAACCAGTCCATGCCAGTCGTCCATAGTACATTAAATTGTTCATCGGACATAGCACATATTCATTAAATAATCCTCCTCACTACGGATGCCCCCCCTCACTTAGGGGTCCCTTGTTCACCATCCTCCGTGAAATCAATATCCCGCACAAGAGTGCTACTCTCCTCGCTCCGGGCCCATAACTCGTGGGGGTAGCTATACTTGAGCTGTATCCG

**WLMQ8 MH730703**

GGCATTTTAACTTAAACTACTTTCTGCATTCTTATGTTGTATAAACTTCATAAAATAACCCTGACACCAACCTACTCATAATACTGCTATGTAATTCGTGCATTACTGCTAGTCAGCATGTATAATATATAGTACTATATATGTTTAACTGTACATAACACATATTATTACATATCAACCTAACATTCTAAACAACATGCTTACAAGCAAGTACCTCGATACAAACCTCAACAGTAACACATAACATGATTCCTTCAAATTCAACTTGTCCTCCTCATGAGTATCAACTAAACCAATCCATGCCAGTCGTCCATAGTACATTAAATTGTTCATCGGACATAGCACATATTCATTAAATAATCCTCCTCACTACGGATGCCCCCCCTCACTTAGGGGTCCCTTGTTCACCATCCTCCGTGAAATCAATATCCCGCACAAGAGTGCTACTCTCCTCGCTCCGGGCCCATAACTCGTGGGGGTAGCTATACTTGAGCTGTATCCG

**WLMQ9 MH730714**

GGCATTCTAACTTAAACTACTTTCTGCATTCTTATGTTGTATAAACTTCATAAAATAACCCTGACACCAACCTACTCATAATACTGCTATGTAATTCGTGCATTACTGCTAGCCAGCATGTATAATATATAGTACTATATATGTTTAACTGTACATAACACATATTATTACATATCAACCTAACATTCTAAACAACATGCTTACAAGCAAGTACCTCAATACAAGTCTCAACAGTAACACATAACATGATTCCTTCAAATTCAACTTGTCCTCCTCATGAGTATCAACTAAACCAGTCCATGCCAGTCGTCCATAGTACATTAAATTGTTCATCGGACATAGCACATATTCATTAAATAATCCTCCTCACTACGGATGCCCCCCCTCACTTAGGGGTCCCTTGTTCACCATCCTCCGTGAAATCAATATCCCGCACAAGAGTGCTACTCTCCTCGCTCCGGGCCCATAACTCGTGGGGGTAGCTATACTTGAGCTGTATCCG

**WLMQ10 MH730691**

GGCATTTTAGCTTAAACTACTTTCTGCATTCTTATGTTATATAAACTTCATAAAATAACCCTGACACCAACCTACTCATAATACTACTATGTAATTCGTGCATTACTGCTAGCCAACATGTATAATATATAGTACTATATATGCTTAACTGTACATAACACATATCATTACATATCAACTTAACATCCTAAACAGCATGCTTACAAGCAAGTACTTTAATACAGACCTCAACAGTAACACATAACACGATCTCTCCAAATTCAACTTGTCCCCCCCATGGATATCAACCAAACCAGTCCATGCCAGTCGTCCATAGTACATTAGATTGTTCATCGGACATAGCACATATTCATTAAATAATCCTCCTCACCACGGATGCCCCCCCTCACTTAGGAATCCCTTGTTCACCATCCTCCGTGAAATCAATATCCCGCACAAGAGTGCTACTCTCCTCGCTCCGGGCCCATAACTCGTGGGGGTAGCTATACTTGAGCTGTATCCG

**WLMQ11 MH730711**

GGCATTCTAACTTAAACTACTTTCTGCATTCTTATGTTGTATAAACTTCATAAAATAACCCTGACACCAACCTACTCATAATACTGCTATGTAATTCGTGCATTACTGCTAGTCAGCATGTATAATATATAGTACTATATATGTTTAACTGTACATAACACATATTATTACATATCAACCTAACATTCTAAACAACATGCTTACAAGCAAGTACCTCAATACAAACCTCAACAGTAACACATAACATGATTCCTTCAAATTCAACTTGTCCTCCTCATGAGTATCAACTAAACCAATCCATGCCAGTCGTCCATAGTACATTAAATTGTTCATCGGACATAGCACATATTCATTAAATAATCCTCCTCACTACGGATGCCCCCCCTCACTTAGGGGTCCCTTGTTCACCATCCTCCGTGAAATCAATATCCCGCACAAGAGTGCTACTCTCCTCGCTCCGGGCCCATAACTCGTGGGGGTAGCTATACTTGAGCTGTATCCG

***M. m. brevicaudus* MH730679**

GGCATTCTAACTTAAACTACTTTCTGCATTCTTATGTTGTATAGGCTCCATAAAATAACCCTGACACCAACTTACCCATAATACTACTATGTAATTCGTGCATTACTGCTAGCCAACATGTATAATATATAGTACTATATATGCTTAACTGTACATAACACATATCATTACATATCAACTTAACATCCTAAACAACATGCTTACAAGCAAGTACTCTAATGCAAGTCTCAACAGTAATACATGACATGACTCCTTCAAACTTGACTTGTTTTCCTCATGAATATCAACTAAACCAGTCCATGCCAGTCGTCCATAGTACATTAAGTTGTTCATCGGACATAGCACATATTTATTAAATAATCCTCCTCACCACGGATGCCCCCCCTCACTTAGGAATCCCTTGTTCACCATCCTCCGTGAAATCAATATCCCGCACAAGAGTGCTACTCTCCTCGCTCCGGGCCCATAACTCGTGGGGGTAGCTATACTTGAACTGTATCCG

***M. m. vestita* MH730684**

GGTATTCTAACTTAAACTACTTTCTGCATTCTTATGTTGTATAAGCCTCATAAAATAACCCTAACACCAACCTACTCATAATATTACTATGTAATTCGTGCATTACTGCTAGCCAACATGTATAATATATAGTACTATATATGCTTAACTGTACATAACACATATCATTACATATCAACCCAACATTCTAGACAACATGCTTACAAGCAAGTACTCTAATATAAACTCCAACAGTAATACATGACATGACCTTTTCAAACTTGATCCGTCCTCCTCATAGATACCAACCGAACCAGCCCATGCCAGTCGTCCATAGTACATTAAATCGTTCATCGGACATAGTACATATTCATTAAATAATCCTCCTCACCACGGATGCCCCCCCTCACTTAGGAGTCCCTTGTTCACCATCCTCCGTGAAATCAATATCCCGCACAAGAGTGCTACTCTCCTCGCTCCGGGCCCATAACTCGTGGGGGTAGCTATACTTGAATTGTATCCG

***M. m. tcheliensis* MH730700**

GGCATTCTAACTTAAACTACTTTCTGCATTCTTATGTTGTATAAGCTTCATAAAATAACCCTGACACCAACCTACTCATAATACTACTATGTAATTCGTGCATTACTGCTAGCCAGCATGTATAATATATAGTACTATATATGTTTAACTGTACATAACACATATCATTACATATCAACTTAACATTCCAAACAACATGCTTACAAGCAAGTACTTTGATACAGACCCCAACAGTAACACATAACACGATCTCTCCAAATTTAACTTGTTCTCCTCATGGATATCAACTAAACCAGTCCATGCCAGTCGTCCATAGTACATTAAATTGTTCATCGGACATAGCACATATTCATTAAATAATCCTCCTCACCACGGATGCCCCCCCTCACTTAGGAATCCCTTGCTCACCATCCTCCGTGAAATCAATATCCCGCACAAGAGTGCTACTCTCCTCGCTCCGGGCCCATAACTCGTGGGGGTAGCTATACTTGAGCTGTATCCG

***M. m. littoralis* MH730728**

GGCATTCTAACTTAAACTACTTTCTGCATTCTTATGTTGTATAAACTTCATAAAATAACCCTGACACCAACCTACTCATAATACTGCTATGTAATTCGTGCATTACTGCTAGTCAGCATGTATAATATATAGTACTATATATGTTTAACTGTACATAACACATATTATTACATATCAACTTAACATTCTAAACAACATGCTTACAAGCAAGTACCTTAATACAAACCTCAACAGTAGTACATAGCATGATTCCTTCAAATTCAACTTGTCCTCCTCATGAGTATCAACTAAACCAGTCCATGCCAGTCGTCCATAGTACATTAAATCGTTCATCGGACATAGCACATATTCATTAAATAATCCTCCTCACCACGGATGCCCCCCCTCACTTAGGAGTCCCTTGTTCACCATCCTCCGTGAAATCAATATCCCGCACAAGAGTGCTACTCTCCTCGCTCCGGGCCCATAACTCGTGGGGGTAGCTATACTTGAGCTGTATCCG

***M. m. lasiotis* MH730749**

TACTTTCTGTATTCTTATGTTGCACAACTTCATAAAATAACCCTAACATTCAACCTACCCATAATATTACTATGTAATTCGTGCATTACTGCTAGCCAACATGTATAATATATAGTACTATATATGCTTAACTGTACATAACACATACCATTACATATCAACTCAACATCCTAGACAACATGCTTACAAGCAAGTACTCTAATACAAGTTCCAACAGTAGTACATAACATGACCTCTCCAAATTCAATTCACTTCCTCATGGATACCAACCGAACCAGTCCATGCCAATCGTCCATAGTACATTAAATCGTTCATCGGACATAGCACATATTCATTAAATAATCCTCCTCACCACGGATGCCCCCCCTCACTTAGGAGTCCCTTGTTCACCATCCTCCGTGAAATCAATATCCCGCACAAGAGTGCTACTCTCCTCGCTCCGGGCCCATAACTCGTGGGGGTAGCTATACTCGAGCTGTATCCG
