# Supplementary figures and images for: Genetic diversity and phylogenetic analyses of 11 cohorts of captive rhesus macaques from Chinese zoos
Source: PeerJ. 2019 May 29;7:e6957. doi: 10.7717/peerj.6957 (PMC6545102; doi:10.7717/peerj.6957)

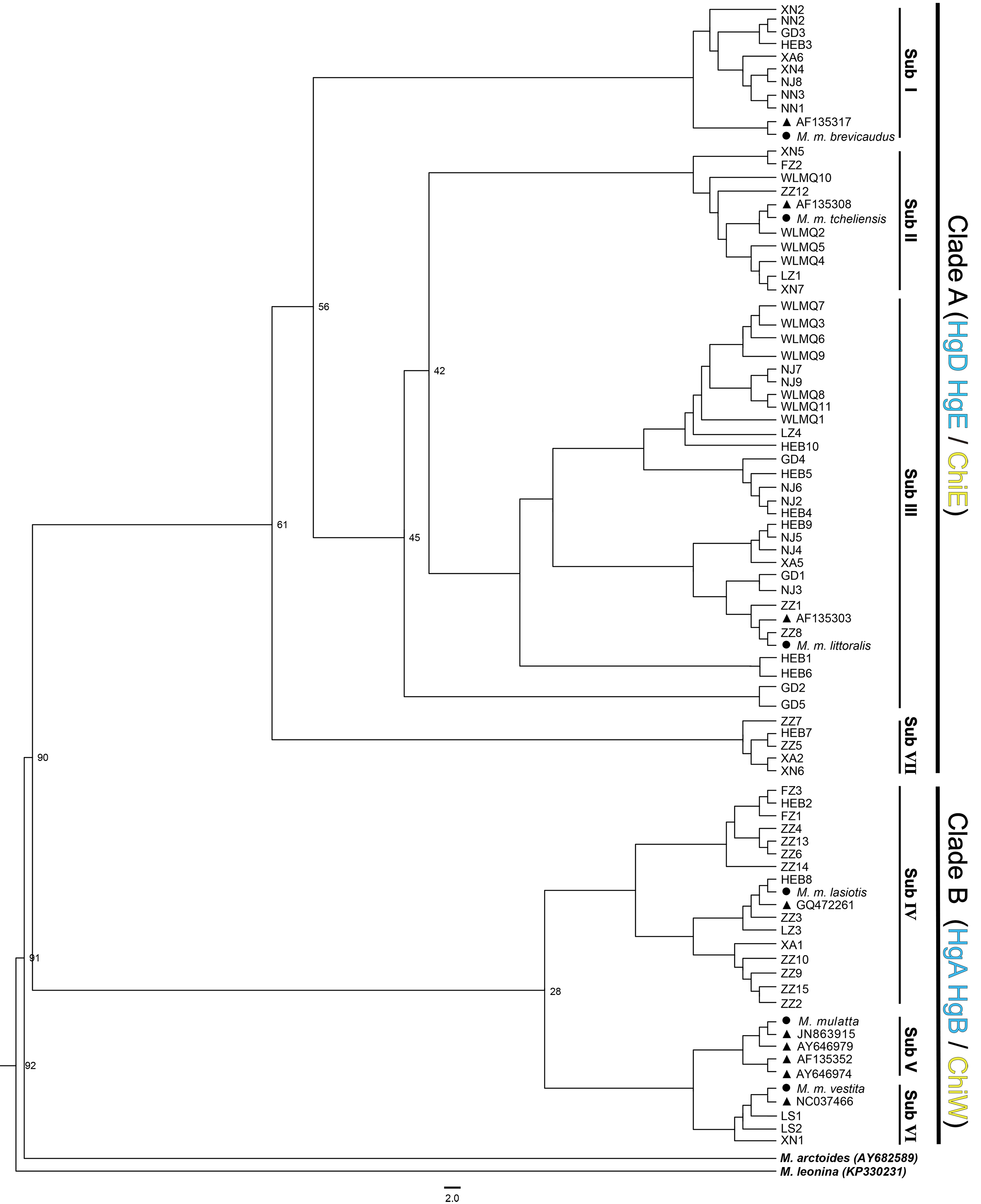

Supplement: Supplemental Information 2 — ● represents the Chinese subspecies based on five wild sequences except M. mulatta, which is represented by one of the Yunnan sequences from NCBI. ▲ represents the other nine wild sequences obtained from NCBI, which originated from Hainan Island, Henan, Guangxi, Sichuan, Yunnan, and Tibet. The northern pig-tailed macaque (M. leonina) and the stump-tailed macaque (M. arctoides) were used as outgroups. Numbers at tree nodes are assigned probabilities, and haplogroup designations are identified by vertical bars at the right. Sub I–VII represents subclades I–VII. The blue labels, HgA, HgB, HgD, and HgE, represent haplogroups A, B, D, and E as defined by Li et al. (2011). The yellow labels, ChiE and ChiW, represent haplogroups defined by Smith & McDonough (2005). ZZ1∼15, FZ1∼2, LZ1∼5, NJ1∼11, XA1∼6, XN1∼7, HEB1∼10, LS1∼2, NN1∼4, GD1∼5, WLMQ1∼11 represent haplotypes from ZZ, FZ, LZ, NJ, XA, XN, HEB, LS, NN, GD and WLMQ zoo, respectively. [file peerj-07-6957-s002.png]

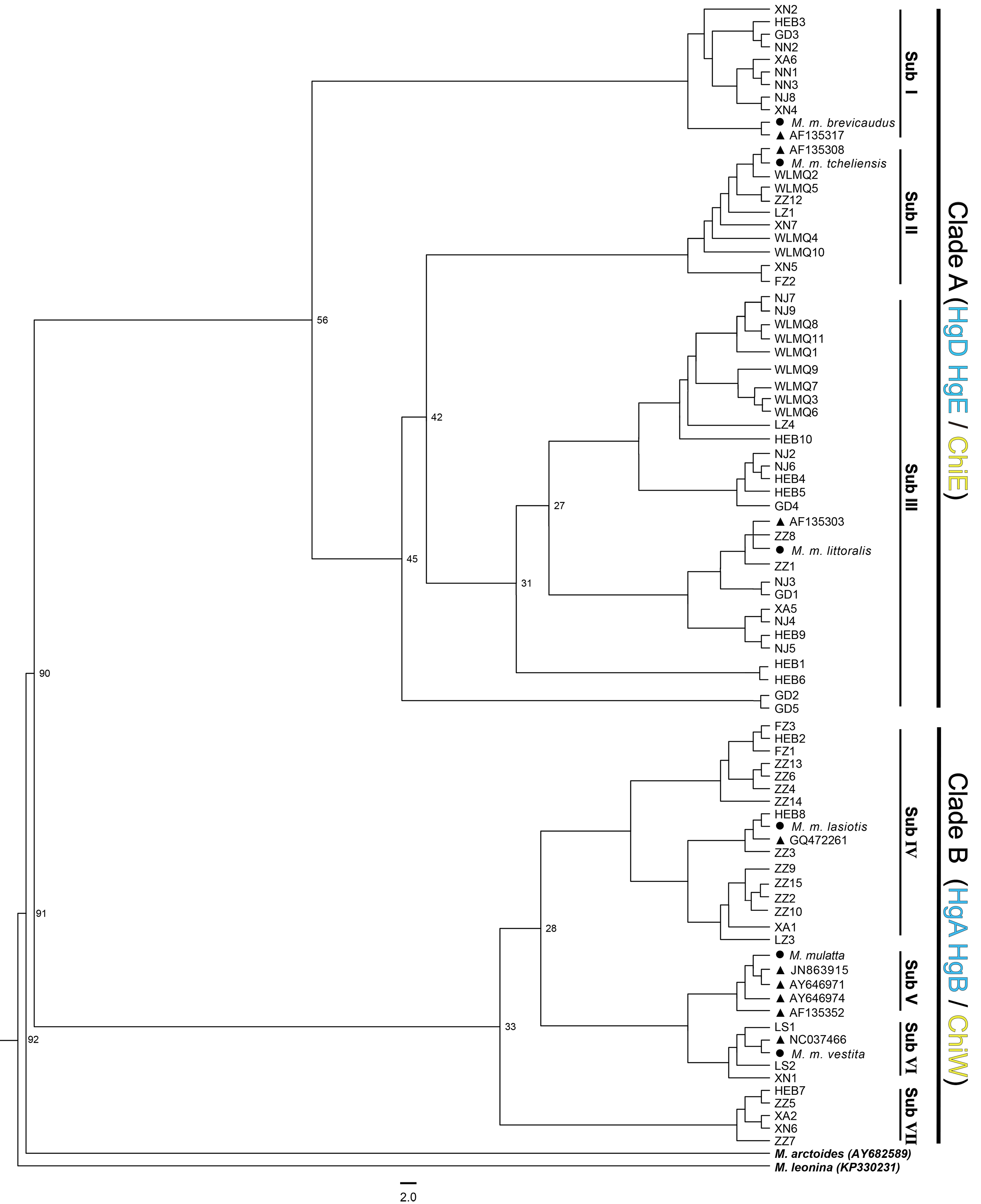

Supplement: Supplemental Information 3 — ● represents the Chinese subspecies based on five wild sequences except M. mulatta, which is represented by one of the Yunnan sequences from NCBI. ▲ represents the other nine wild sequences obtained from NCBI, which originated from Hainan Island, Henan, Guangxi, Sichuan, Yunnan, and Tibet. The northern pig-tailed macaque (M. leonina) and the stump-tailed macaque (M. arctoides) were used as outgroups. Numbers at tree nodes are assigned probabilities, and haplogroup designations are identified by vertical bars at the right. Sub I–VII represents subclades I–VII. The blue labels, HgA, HgB, HgD, and HgE, represent haplogroups A, B, D, and E as defined by Li et al. (2011). The yellow labels, ChiE and ChiW, represent haplogroups defined by Smith & McDonough (2005). ZZ1∼15, FZ1∼2, LZ1∼5, NJ1∼11, XA1∼6, XN1∼7, HEB1∼10, LS1∼2, NN1∼4, GD1∼5, WLMQ1∼11 represent haplotypes from ZZ, FZ, LZ, NJ, XA, XN, HEB, LS, NN, GD and WLMQ zoo, respectively. [file peerj-07-6957-s003.png]

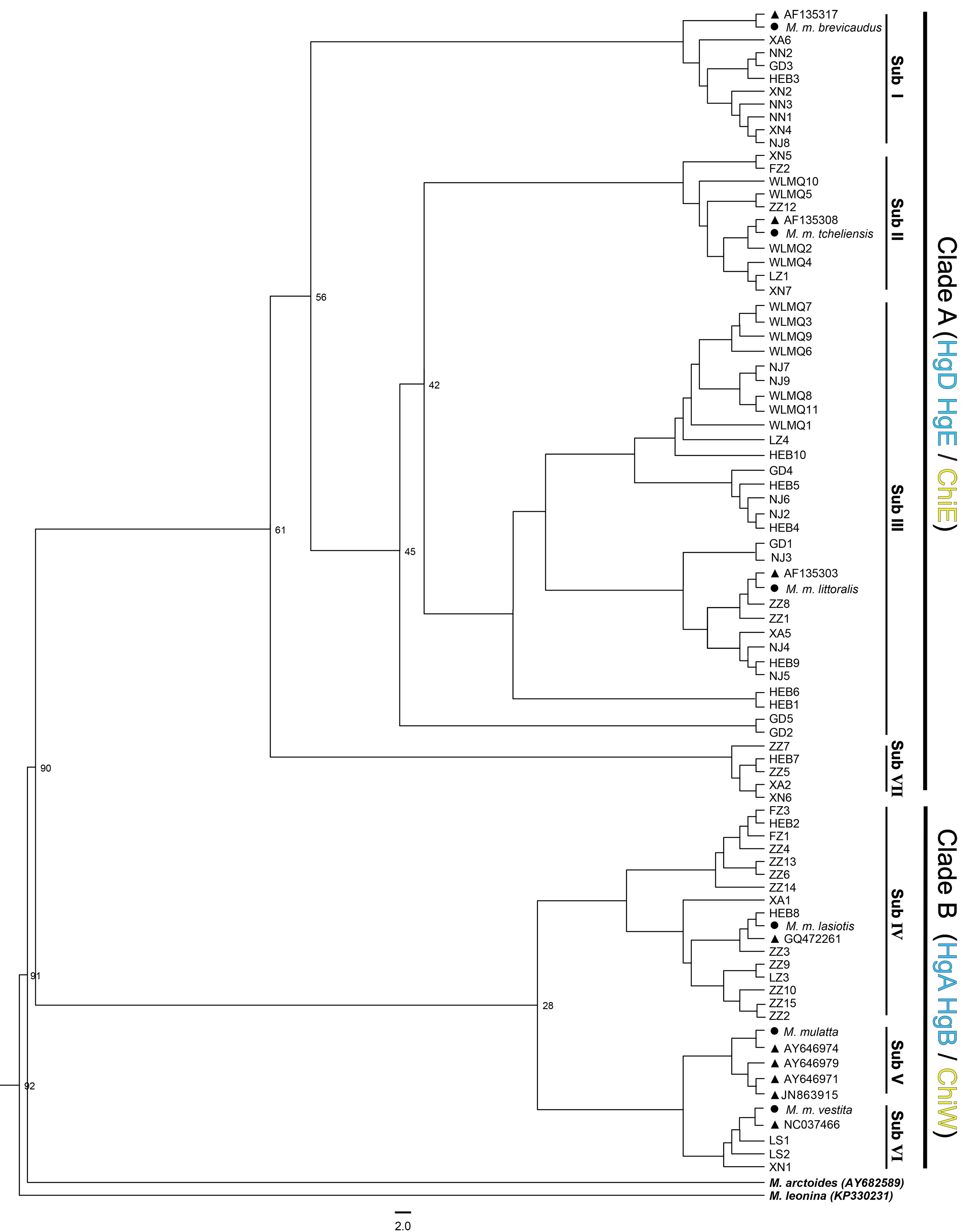

Supplement: Supplemental Information 4 — ● represents the Chinese subspecies based on five wild sequences except M. mulatta, which is represented by one of the Yunnan sequences from NCBI. ▲ represents the other nine wild sequences obtained from NCBI, which originated from Hainan Island, Henan, Guangxi, Sichuan, Yunnan, and Tibet. The northern pig-tailed macaque (M. leonina) and the stump-tailed macaque (M. arctoides) were used as outgroups. Numbers at tree nodes are assigned probabilities, and haplogroup designations are identified by vertical bars at the right. Sub I–VII represents subclades I–VII. The blue labels, HgA, HgB, HgD, and HgE, represent haplogroups A, B, D, and E as defined by Li et al. (2011). The yellow labels, ChiE and ChiW, represent haplogroups defined by Smith & McDonough (2005). ZZ1∼15, FZ1∼2, LZ1∼5, NJ1∼11, XA1∼6, XN1∼7, HEB1∼10, LS1∼2, NN1∼4, GD1∼5, WLMQ1∼11 represent haplotypes from ZZ, FZ, LZ, NJ, XA, XN, HEB, LS, NN, GD and WLMQ zoo, respectively. [file peerj-07-6957-s004.png]
