# Supplementary material for: Genetic diversity and phylogenetic analyses of 11 cohorts of captive rhesus macaques from Chinese zoos
Source: PeerJ. 2019 May 29;7:e6957. doi: 10.7717/peerj.6957 (PMC6545102; doi:10.7717/peerj.6957)
Supplement: Supplemental Information 5 — The parenthesized numbers are sample quantities and the underlined represent shared haplotypes between zoos. 1∼7 represent haplotypes shared by zoos. [file peerj-07-6957-s005.docx]

| ZZ(38) | FZ(13) | LZ(19) | NJ(14) | XA(7) | XN(38) | HEB(24) | LS(17) | NN(9) | GD(12) | WLMQ(19) |
| --- | --- | --- | --- | --- | --- | --- | --- | --- | --- | --- |
| ZZ1(1) | FZ1(7) | LZ1(5) | **NJ1(1)^3^** | XA1(1) | XN1(5) | **HEB1(5)^5^** | **LS1(5)^2^** | NN1(3) | **GD1(7)^7^** | WLMQ1(3) |
| **ZZ2(1)^1^** | FZ2(5) | **LZ2(4)^2^** | NJ2(1) | XA2(1) | XN2(10) | HEB2(2) | LS2(12) | NN2(3) | **GD2(2)^4^** | WLMQ2(1) |
| ZZ3(8) | FZ3(1) | LZ3(7) | NJ3(1) | **XA3(1)^1^** | **XN3(5)^7^** | HEB3(2) |  | NN3(2) | GD3(1) | WLMQ3(2) |
| ZZ4(5) |  | LZ4(3) | NJ4(1) | **XA4(2)^6^** | XN4(7) | HEB4(2) |  | **NN4(1)^4^** | GD4(1) | WLMQ4(2) |
| ZZ5(3) |  |  | NJ5(1) | XA5(1) | XN5(3) | HEB5(1) |  |  | GD5(1) | WLMQ5(2) |
| ZZ6(4) |  |  | NJ6(2) | XA6(1) | XN6(1) | **HEB6(3)^6^** |  |  |  | WLMQ6(2) |
| ZZ7(2) |  |  | NJ7(1) |  | XN7(7) | HEB7(3) |  |  |  | **WLMQ7(2)^3^** |
| ZZ8(1) |  |  | NJ8(4) |  |  | HEB8(1) |  |  |  | WLMQ8(1) |
| ZZ9(1) |  |  | NJ9(2) |  |  | HEB9(4) |  |  |  | WLMQ9(2) |
| ZZ10(2) |  |  |  |  |  | HEB10(1) |  |  |  | WLMQ10(1) |
| **ZZ11(2)^5^** |  |  |  |  |  |  |  |  |  | WLMQ11(1) |
| ZZ12(1) |  |  |  |  |  |  |  |  |  |  |
| ZZ13(1) |  |  |  |  |  |  |  |  |  |  |
| ZZ14(1) |  |  |  |  |  |  |  |  |  |  |
| ZZ15(5) |  |  |  |  |  |  |  |  |  |  |

**Supplementary Table 1**  Distribution of 69 haplotypes in 11 zoo rhesus macaque populations in China.

Note: The parenthesized numbers are sample quantities and the underlined represent shared haplotypes between zoos. **^1~7^** represent haplotypes shared by zoos.
